# Quartz Crystal Microbalance Electronic Interfacing Systems: A Review

**DOI:** 10.3390/s17122799

**Published:** 2017-12-04

**Authors:** Abdulrahman Alassi, Mohieddine Benammar, Dan Brett

**Affiliations:** 1 Department of Electrical Engineering, Qatar University, Doha 2713, Qatar; 2 Department of Chemical Engineering, University College London, London WC1E 6BT, UK; d.brett@ucl.ac.uk

**Keywords:** quartz crystal microbalance, BVD model, impedance analyzers, QCM oscillators, Phase-Locked-Loop, QCM-D, Contactless QCM, Phase-Mass QCM, high-temperature microbalance

## Abstract

Quartz Crystal Microbalance (QCM) sensors are actively being implemented in various fields due to their compatibility with different operating conditions in gaseous/liquid mediums for a wide range of measurements. This trend has been matched by the parallel advancement in tailored electronic interfacing systems for QCM sensors. That is, selecting the appropriate electronic circuit is vital for accurate sensor measurements. Many techniques were developed over time to cover the expanding measurement requirements (e.g., accommodating highly-damping environments). This paper presents a comprehensive review of the various existing QCM electronic interfacing systems. Namely, impedance-based analysis, oscillators (conventional and lock-in based techniques), exponential decay methods and the emerging phase-mass based characterization. The aforementioned methods are discussed in detail and qualitatively compared in terms of their performance for various applications. In addition, some theoretical improvements and recommendations are introduced for adequate systems implementation. Finally, specific design considerations of high-temperature microbalance systems (e.g., GaPO_4_ crystals (GCM) and Langasite crystals (LCM)) are introduced, while assessing their overall system performance, stability and quality compared to conventional low-temperature applications.

## 1. Introduction

Piezoelectric crystals are widely considered as an essential component in contemporary electronic applications. Their operating principle relies on the piezoelectric effect that was first discovered by the Curie brothers in 1880 [1]. These crystals may be used as mass sensors, known as the Quartz Crystals Microbalance (QCM), conventionally through sandwiching a thin crystal between two conducting electrodes. That is, the crystal’s natural oscillation frequency is altered in response to small deposited mass adlayers. Sauerbrey introduced an expression relating the detectable shift in the crystal’s resonance frequency ($\Delta f_{m}$) to the mass deposited (Δm) in gaseous/vacuum media in 1959 [2]. This resonance frequency shift is also dependent on the unloaded crystal’s resonant frequency ($f_{o}$), its odd harmonic overtone (N=1,3,…), the crystal’s effective surface area ($A_{e}$), its density ($\rho_{q}$) and the shear modulus of the crystal’s material ($\mu_{q}$).

(1) $$\frac{\Delta f_{M}}{f_{o}}=-\frac{2Nf_{o}\Delta m}{A_{e}\sqrt{\mu_{q}\rho_{q}}}$$

Equation (1) represents relative resonance frequency changes with respect to mass variations, depicting accurate responses for thin and rigid uniform films [3,4], by treating the deposited mass adlayer as an extension to the crystal’s thickness. Consequently, the thickness extension approximation loses its validity for frequency shifts exceeding 2% of unloaded crystal resonance [5,6]. Many applications are limited to lower frequency variations that are typically measured by part-per-million (*ppm*). Yet, an extended model based on the ‘Z-Match’ method is presented in [7] to overcome this limitation. Unlike Equation (1), this model considers the acoustic properties (e.g., impedance) of the deposited rigid adlayer rather than treating it as a crystal material extension. Thus, extending the practical mass-frequency interpretation range to 15% of $f_{o}$ [7] and up to 40% for some applications [8,9].

Nonetheless, other factors (e.g., temperature) may also affect the QCM frequency stability. Conventionally, AT-cut quartz crystals are adopted for QCM sensors due to their near-zero temperature coefficient around room temperature. Typically, a 1 ppm/° change in oscillation frequency is observed for AT-cut quartz within the 10 to 50 °C range [10]. However, temperature-induced frequency variation ($\Delta f_{T}$) is observed when the sensor is required to operate under elevated temperature environments, where this shift should be compensated for accordingly [10,11].

In the early 1980s, the use of QCM sensors was significantly expanded, especially in biosensing applications, by considering operation in liquid media [12,13]. The well-known work of Kanazawa and Gordon [14] led to the development of Equation (2) which governs resonance frequency-shift of QCM when operated in liquids.

(2) $$\frac{\Delta f_{L}}{f_{o}}=-\sqrt{\frac{f_{o}\rho_{L}\eta_{L}}{\pi \rho_{q}\mu_{q}}}$$

where $\rho_{L}$ and $\eta_{L}$ are the damping medium’s density and viscosity, respectively. Thus, the overall resonance frequency shift in microbalance applications mainly incorporates a combined effect of mass, temperature and viscosity, as summarized in Equation (3).

(3) $$\Delta f\approx \Delta f_{M}+\Delta f_{T}+\Delta f_{L}$$

The aforementioned effects are more representative for most microbalance applications, yet, other spurious factors (e.g., pressure and stress) may also cause additional minor frequency shifts that should be taken into account when present [15]. Generally, QCMs are used as chemical and biological sensors for mass, viscosity, temperature and humidity measurements, in addition to recently being used as gas sensors in some applications [16,17,18,19,20,21,22,23,24,25,26,27,28,29,30,31,32,33,34,35]. Quantitatively, 569 published works tackled molecular recognition studies based on QCM sensors from 2001 and 2005 [36], compared to 857 works focusing on QCM’s chemical and biochemical applications between 2006–2009 [37]. This huge number indicates the active nature of QCM applications and the necessity of identifying appropriate electronic interfacing circuit for the intended application.

It is important to note that Equations (1) and (2) indicate an increase in the measurement sensitivity as a function of (*f_o_*). However, a practical maximum limit is set to *f_o_* as it is inversely proportional to crystal’s thickness and its quality factor [38,39]; where the latter should ideally be maximized. Accordingly, the development of High Fundamental Frequency (*HFF*) QCM resonators operating at 150 MHz was recently reported [40]. Electrodeless QCM biosensors operating at a fundamental resonance frequency of 170–180 MHz are also newly reported [41]. Specifically, recent works introduced wireless-electrodeless QCM operation to increase the resonator’s sensitivity and detection limits [42,43,44,45,46]. This is because parallel electrodes deteriorate the overall QCM sensitivity and range since they are also considered as deposited adlayers [42]. The effect of parallel electrodes geometry and distribution on the resonator’s sensitivity is recently discussed in [47], in addition to the effect of adlayers radial distribution. That is, QCM sensitivity is found to be the highest at the electrodes center and decreases exponentially as the radial distance increases [47,48]. A dynamic extended model is thus introduced in [47] to account for the aforementioned parameters, especially when geometrically symmetric, uniform adlayers are unexpected.

Other alternatives for applications requiring elevated sensitivities are the Film Bulk Acoustic Resonators (*FBAR*) sensors, with fundamental resonance frequencies within the GHz range [49]. Higher mass sensitivities are achieved by these sensors, at the expense of higher noise levels, establishing a practical tradeoff. A comprehensive comparison between these sensors is found elsewhere [41,50].

The discussion above has been focused on conventional QCM applications with temperature ranges within the quartz low-temperature coefficient regions; such applications make up for the majority of crystal microbalance applications. Yet, QCM systems have also been developed for use under extremely low temperatures (e.g., space exploration applications) [51,52]. On the other side of the spectrum, some applications require stable operation at higher temperatures. Quartz maximum operating temperature as a piezoelectric material is limited by its phase-transition, Curie temperature, of 573 °C [53]. In contrast, other types of novel, quartz-like, crystals are shown to possess similar piezoelectric characteristics to the conventional quartz for QCM applications at higher temperatures [53,54]. For instance, the Y-11.1° cut gallium orthophosphate (GaPO_4_) based crystals are able to maintain stable operation under temperatures as high as 900 °C with a linearly decreasing temperature coefficient, reaching 3 $\mathrm{Hz}\;{{}^{\circ}C}^{-1}$ at 450 °C, based on the manufacturing specifications [53]. The phase-transition temperature of this material is reported around 920 °C, in addition to its higher mechanical quality factor (*Q*), indicating its superiority over quartz for such applications.

**Figure 1:**
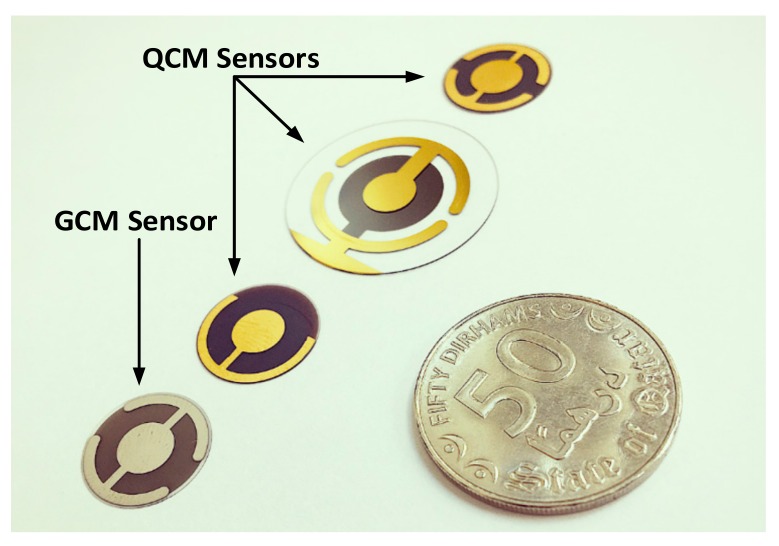
Sample commercial GCM and QCM sensors.

The utilization of GaPO_4_ based material in sensing applications, known as GCM, is reported in several works [53,54,55,56,57,58]. Figure 1 shows different types of QCM sensors with various sizes and Au/Cr-electrodes distribution, in addition to a *Pt*-electrodes coated R-30 GCM sensor from *PiezoCryst* for comparison. Finally, a comprehensive review of high-temperature crystal types and characteristics, including the emerging Langasite based LCM sensors, is found elsewhere [59].

Subsequently, the main focus of this paper is on analyzing the existing QCM electronic interfacing sensors based on their wider-utilization in literature. Some interfacing techniques are based on the described resonance, others are based on direct impedance or phase shift measurements that are representative of resonance shifts. Although QCM and GCM characterization is somewhat similar, a separate section of this paper is dedicated to GCM and high-temperature microbalance applications, with relevant design considerations and recommendations.

The structure of this paper is as follows: electrical modeling of crystal resonators is first introduced. After that, QCM electronic measurement systems and techniques are thoroughly analyzed in the following order: (1) Impedance-based measurements. (2) Electronic oscillator circuits, in addition to their enhanced *lock-in* oscillator systems. (3) Impulse Excitation/Decay technique (QCM-D). (4) QCM Phase-mass technique. High-temperature microbalance systems design considerations and recommendations are then discussed in terms of their electronic interfacing circuits and physical aspects. A qualitative comparison of the different electronic characterization systems is then presented, detailing the advantages/disadvantages of each category for different applications and operating mediums, followed by concluding remarks.

## 2. Electrical Modeling of QCM Resonators

**Figure 2:**
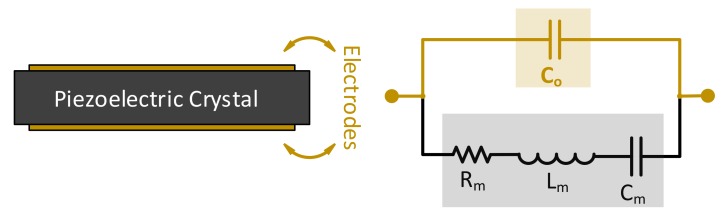
(**Left**) microbalance crystal diagram. (**Right**) Corresponding BVD electrical model.

From the electrical point of view, piezoelectric thickness shear mode (*TSM*) crystals are modeled as acoustic transmission lines [60]. Yet, simplified versions of this model have been developed over time. Namely, the Lumped Elements Model (*LEM*), which models the loading effect as a complex impedance $Z_{L}$ series to the motional Butterworth-Van-Dyke (*BVD*) branch [60]. Consequently, unloaded QCM sensors are electrically modeled, near resonance, using the generic BVD circuit model, consisting of a series *RLC* motional arm, with $\left(C_{m}\right)$ representing the energy stored per oscillation, $\left(R_{m}\right)$ representing the energy loss, damping, per oscillation and $\left(L_{m}\right)$ as the inertial element related to vibrating mass of the crystal unit [61]. Another capacitor $\left(C_{o}\right)$ is added across the motional arm to account for the electrodes capacitance with the piezoelectric crystal acting as the dielectric material, where its capacitance value is dependent on the crystal geometry and is typically in the *pF* range. The simple BVD circuit model is shown in Figure 2 and related to the sensor composition.

**Figure 3:**
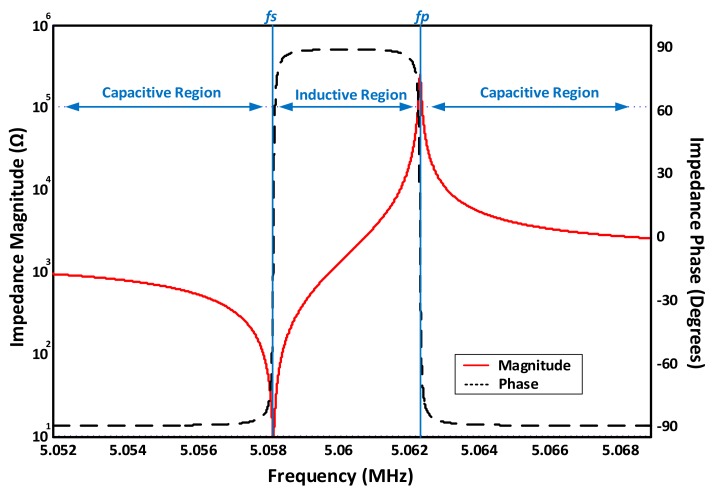
Impedance amplitude and phase plots around resonance frequency for a typical high quality, lightly loaded, 5 MHz crystal.

As seen from the circuit in Figure 2, two fundamentals resonance frequencies may be achieved at the resonator’s minimum and maximum impedance points. Namely, series ($f_{s})$ and parallel resonance ($f_{p}$). Series resonance is mainly related to the crystal itself and more representative of its behavior. Yet, the series minimum impedance frequency ($f_{s}$) is different from the true motional series resonant frequency (*MSRF*), which is only dependent on $L_{m}C_{m}$ resonance and is considered to be the frequency of interest for most QCM applications, representing actual piezoelectric crystal resonance. On the other hand, the parallel resonance frequency is mainly attributed to the parallel capacitor resonance with the motional branch. Both frequencies are obtained as in Equations (4) and (5), serving as accurate approximations for high quality resonators as depicted in Figure 3.

(4) $$f_{s}\approx \frac{1}{2\pi \sqrt{L_{m}C_{m}}}$$

(5) $$f_{P}\approx \frac{1}{2\pi \sqrt{L_{m}\frac{C_{m}C_{o}^{*}}{C_{m}+C_{o}^{*}}}}$$

where $C_{o}^{*}=C_{o}+C_{ext}$ assumes a connected resonator to external apparatus and is replaced by $C_{o}$ in case of bare-electrodes, as discussed above. Equation (4) resembles MSRF, where $f_{s}=MSRF$ only if $R_{m}=0$ or if the sensor is electrodeless. The simple BVD components values may be theoretically calculated as in Equation (6) for a given resonance frequency, or one of its harmonic overtones [62].

(6a) $$C_{o}=\frac{\epsilon_{p}A}{h}$$

(6b) $$C_{m}=\frac{8K_{o}^{2}C_{o}}{{\left(N\pi \right)}^{2}}$$

(6c) $$L_{m}=\frac{1}{\omega_{MSRF}^{2}C_{m}}$$

(6d) $$R_{m}=\frac{\eta_{p}}{c_{p}C_{1}}{\left(\frac{\omega }{\omega_{MSRF}}\right)}^{2}$$

where $\omega \;\mathrm{and}\;\omega_{MSRF}$ are the operating and *MSRF* frequencies, respectively. Equation (6) may be inaccurate under certain conditions [38]; thus, practical characterization techniques may be alternatively used (e.g., based on impedance analysis) as will be discussed further down. Figure 3 also demonstrates the different operating regions of a high-quality QCM sensor, near resonance, based on a practical case of 5-MHz QCM crystals [9]. The BVD parameters are provided as: $L_{m}=30\;\mathrm{mH},\;C_{m}=33\;\mathrm{fF},\;R_{m}=10\;Ω$ and $C_{o}^{*}=20\;\mathrm{pF}$. Sharp phase-transition between inductive and capacitive regions is observed at resonance from −90° to 90° and back. The aforementioned approximations are accurately valid for QCM applications in vacuum/gas phase, where the medium’s damping is mostly negligible [63] and the deposited uniform films are modeled through BVD as a small inductance, series to the motional arm, causing the resonance curves in Figure 3 to slightly shift to the lower-frequency side.

**Figure 4:**
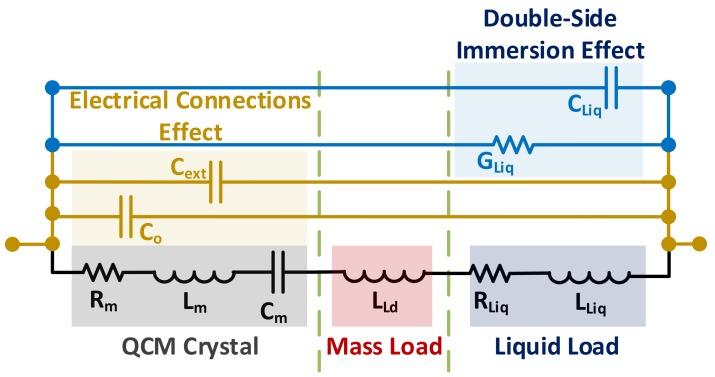
Detailed BVD model of a loaded QCM crystal in liquid.

On the other hand, liquid-phase applications are associated with significant damping of the QCM resonator due to viscous loading, in addition to further shifts in resonance frequency. Such changes are modeled by adding series $R_{liq}$ and $L_{liq}$ components to the motional branch of the equivalent circuit. Single-sided liquid immersion is used for most in-liquid applications. Yet, double-side immersion is still adopted by some works [39,64]. For the latter case, the liquid conductivity, modeled by $G_{liq}$ and permittivity, modeled by $C_{liq}\;$ are taken into account when modeling the resonator [39,65] and are omitted otherwise. Intuitively, using the sensor in highly-conductive liquids under complete-immersion conditions would short the sensor output. Figure 4 illustrates the detailed, loaded BVD model, near resonance, for a QCM resonator.

For the standard 5 MHz crystal characterized in Figure 3, a single-side immersion in pure water corresponds to increasing the overall motional resistance $R_{s}^{T}=R_{m}+R_{liq}$ to 400 Ω (*liquid-induced resistance* $R_{liq}$ = 390 Ω), in addition to a liquid-induced inductance $L_{liq}$ = 8.48 µH [9], with $L_{s}^{T}=L_{m}+L_{LD}+L_{liq}$. The other parameters ideally maintain their constant values. Significant increase in the total motional arm resistance, compared to the minimal inductance change, negatively affects the QCM quality factor (*Q*), which represents the ratio of the stored to lost energy for each crystal oscillation [66]. The basic formula for determining *Q* is expressed as a ratio of the resonator’s equivalent inductive reactance at resonance to its equivalent resistance, as in Equation (7).

(7) $$Q=\frac{j\omega_{o}L_{s}^{T}}{R_{s}^{T}}$$

**Figure 5:**
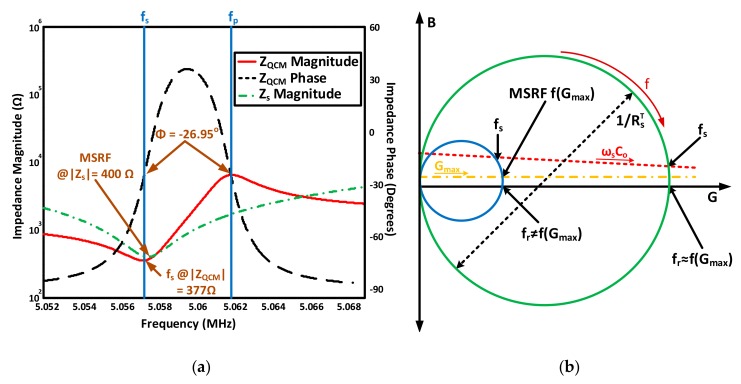
Effect of damping on QCM response. (**a**) Impedance magnitude and phase responses around resonance, for a liquid-loaded, typical 5 MHz QCM crystal; (**b**) QCM admittance locus under different damping conditions.

Practical consequences of *Q* degradation on the QCM frequency response are illustrated in Figure 5a. That is, the minimum motional impedance ($Z_{s}$) point, corresponding to *MSRF* and maximum conductance and represented by Equation (4), occurs after the overall resonator’s minimum impedance point ($Z_{QCM}$), represented by $f_{s}$. Such behavior is mainly influenced by the parallel $R_{s}^{T}C_{o}$ combination near resonance, which is no longer dominated by $R_{s}^{T}$ for damped mediums and considerably affects the points of absolute minimum/maximum impedance and their phase. As a result, series and parallel resonance points occurred at −26.95°, compared to the case of unloaded resonator (i.e., $R_{m}=10\;Ω$), where both resonance frequencies occurred around the *zero-phase* crossing point. In addition, the inductive phase-transition hardly reaches 30° for the given liquid-loading scenario due to the narrow inductive region that, combined with the large damping resistance, prevents the inductive reactance to build-up significantly and dominate the sensor’s overall impedance before resonating with $C_{o}^{*}$.

Subsequently, the total parallel static capacitance $C_{o}^{*}$ negative effects on the measured resonance frequency in viscous mediums require thorough evaluation. The reported Z*_min_* value in Figure 5a was 377 Ω, compared to 400 Ω for the actual $R_{s}^{T}$, indicating a false interpretation of the sensor damping and the corresponding liquid viscosity if the absolute minimum impedance point is taken as references for *MSRF* and $R_{s}^{T}$ estimation. Also, a difference of ~254 Hz between the *MSRF* and the actual detected minimum impedance frequency $f_{s}$ is measured for the given scenario. These variations are for the case of operating in pure water and are amplified significantly for more viscous mediums [9]. Thus, the maximum conductance (*G*) point should be taken as a global reference for accurate *MSRF* estimation instead as it is independent of $C_{o}^{*}$. $R_{s}^{T}$ is then simply calculated as the reciprocal of $G_{max}$.

Figure 5b qualitatively describes the practical implications of the parallel capacitance effect through admittance locus, adopted from [63] with some improvements, where the outer circle represents an unloaded QCM scenario (i.e., low $R_{s}^{T}$) and the inner circle represents a damped resonator scenario (only $R_{liq}$ is considered for simplicity in this scenario, since the goal is to study the combined $C_{o}^{*}$, $R_{liq}$ effect, rather than $L_{liq}$ mass loading). The orange horizontal dashed line represents the maximum conductance trajectory, independent of $C_{o}^{*}$ (i.e., the MSRF line). On the other hand, the slightly inclined red dashed line represents the measured $f_{s}$ at the minimum impedance point. This inclination represents the electrical susceptance (*B: reciprocal of reactance*) behavior of the sensor, where the angular frequency $\omega_{s}$ varies based on the minimum impedance point as in Figure 5b. A recent paper discussed the influence of motional resistance and parallel capacitance variations on the resonator’s series resonance point for liquid-phase applications [67]. Detailed resonance-band characterization may also be reviewed from the following recommended references [39,63,65].

Finally, it should be noted that additional modifications can be introduced to the BVD model based on several additional factors, such as coating the QCM with polymer films, or adding extra components to indicate the viscoelastic behavior of some loaded film. Such adjustments are important for the BVD model accuracy when it is used as part of the sensor characterization, by making it more representative of the actual application [68].

## 3. QCM Electronic Interfacing Systems for Sensing Applications

Several QCM electronic measurements systems exist in literature, varying in their level of complexity, cost, accuracy and portability. These interfacing circuits aim to accurately measure physical variations that are related to relevant physical quantities (e.g., mass ($\Delta L_{m}$), viscosity ($R_{liq}$) and film rigidity (D) variations on the sensor’s surface) based on the specific application requirements. From electrical point of view, the main measurements are related to BVD parameters impedance variations, which may be measured through resonance frequency (Equations (1) and (2)), or phase-shift variations as employed by some techniques, since both quantities are inter-related. The main systems are comprehensively discussed and analyzed in this section, in terms of explaining their working principle, design considerations and practical examples.

### 3.1. Impedance-Analysis Based Measurements

**Figure 6:**
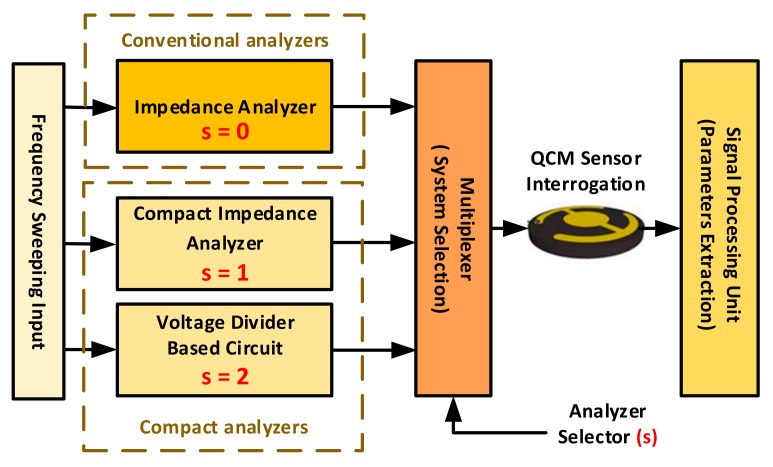
Different types of impedance-based measurement systems in literature.

This technique is traditionally based on expensive and bulky lab-based impedance analyzers, where it has been applied in different applications [69,70,71], including the use of QCM resonators as humidity sensors [31,32,33]. On the other hand, various attempts have been made by researchers to maintain conventional analyzers advantages while developing compact systems. Thus, impedance-based measurements may be divided into two main subcategories; namely, conventional and compact analyzers based. Figure 6 illustrates the basic operation of these different systems. The basic principle is similar to that shown in Figure 3 and Figure 5a, where the sensor is passively interrogated by a frequency-sweeping signal around resonance to analyze its impedance/admittance behavior.

#### 3.1.1. Conventional Impedance Analyzers Based Measurements

For conventional analyzers, conductance curves are generated to determine the maximum conductance, *MSRF*, frequency. Calculating the conductance reciprocal at that frequency results in the total motional resistance $R_{m}$. Repeating this process indefinitely in controlled time intervals and recorded computerized fashion can easily track MSRF and $R_{m}$ variations and relate them to the corresponding Sauerbrey and damping monitoring equations. Simultaneous susceptance (*B*) measurement may also be used to identify $C_{o}^{*}$ by measuring the sensor *B* at *MSRF*. This idea is illustrated mathematically as follows. First, the overall QCM impedance is calculated, as in Equation (8a), the admittance is then obtained as its reciprocal in Equation (8b).

(8a) $$Z=Z_{s}\;//\;Z_{p}=\frac{\left[R_{m}+j\left(\omega L_{m}-\frac{1}{\omega C_{o}^{*}}\right)\right]\left[\frac{1}{j\omega C_{o}^{*}}\right]}{R_{m}+j(\omega L_{m}-\left[\frac{1}{\omega }\left(\frac{C_{m}+C_{o}^{*}}{C_{m}C_{p}}\right)\right]}$$

(8b) $$Y=\frac{1}{Z}=\frac{R_{m}}{R_{m}^{2}+{\left(\omega L_{m}-\frac{1}{\omega C_{m}}\right)}^{2}}+j\left(\frac{\omega L_{m}-\frac{1}{\omega C_{m}}}{R_{m}^{2}+{\left(\omega L_{m}-\frac{1}{\omega C_{1}}\right)}^{2}}+\omega C_{o}^{*}\right)$$

**Figure 7:**
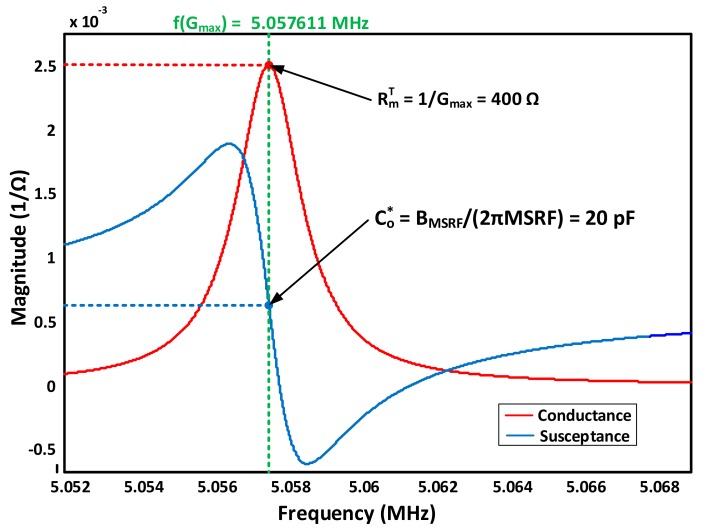
Impedance analyzers characterization principle in measuring BVD parameters for a typical 5 MHz QCM.

At *MSRF*, the expression reduces to Equation (8c) and thus $R_{m}$ and $C_{o}^{*}$ can then be easily obtained as in Figure 7, where the scenario of QCM single-side immersion in pure water with $R_{m}^{T}=400\;\Omega$ is maintained.

(8c) $$Y=\frac{1}{R_{m}}+j\omega_{MSRF}C_{o}^{*}$$

Accordingly, a complete sensor characterization is achieved, where $C_{m}$ and $L_{m}$ may be easily obtained through non-linear curve fitting techniques. The external apparatus capacitance $C_{ext}=C_{o}^{*}-C_{o}$ should be compensated through setup calibration or direct measurement for accurate $C_{o}$ estimation.

The main advantages of *conventional* impedance analyzing QCM characterization networks are identified compared to other techniques. Namely, the sensor is characterized in isolation without the influence of external circuitry, providing significantly more accurate estimations, typically the most accurate measurements compared to any other technique. On the other hand, the conductance curve peak flatness, corresponding to a low quality factor *Q* in highly damped operating conditions, requires the utilization of impedance analyzers supporting high-frequency resolution to ensure extended accuracy [39,72].

In contrast, these systems are disadvantageous in terms of their high cost and bulky size, limiting their practicality to *lab-based* measurements [39,63,72,73]. Additionally, the required sweeping time per reading may be essential for some QCM applications, especially when rapidly changing conditions are expected. Furthermore, ensuring a maximized signal-to-noise (*SNR*) ratio is important for accurate interpretations. Thus, the analyzer selection should take such points into consideration [63,74]. Other electronic characterization techniques based on *lock-in* oscillator concepts were also introduced in literature to overcome the problem for fast QCM applications [75].

**Figure 8:**
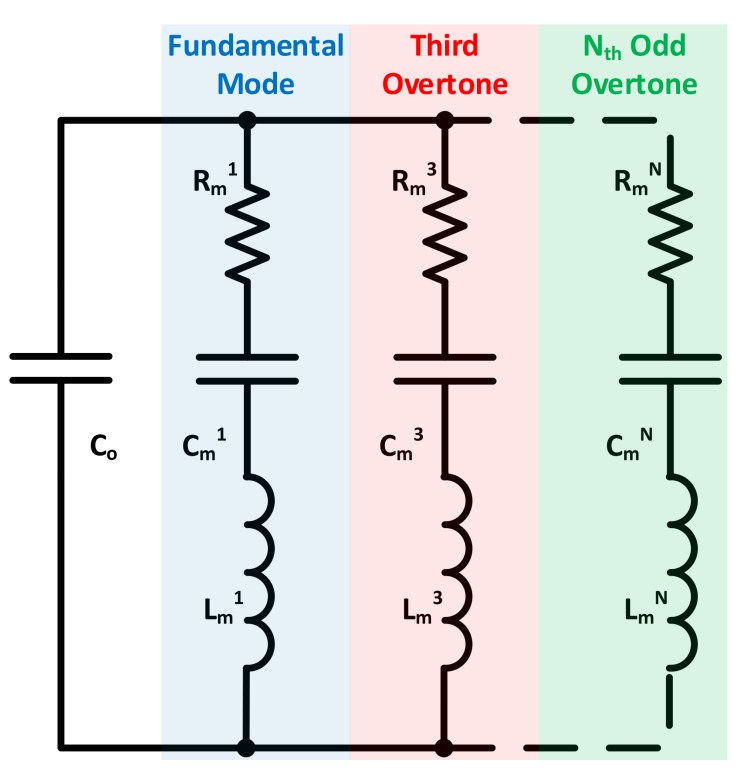
Extended unloaded BVD model, including parallel harmonic overtones branches.

Additionally, conventional frequency-sweeping based impedance analyzers are used in literature to study the QCM behavior at higher order overtones. Only odd harmonics are electrically excited when the sensor is interrogated at the appropriate frequency (i.e., *N* = 1, 3, 5, …). These harmonic overtones are modeled electrically as parallel motional branches (Figure 8). Only the branch corresponding to the interrogating resonance harmonic/overtone is excited, whereas the others are essentially seen by the source as open-circuits. Motional branch parameters (i.e., $R_{m},\;C_{m}\;\mathrm{and}\;L_{m}$) are thus dependent on the excited overtone [76], which is also observed from Equation (6).

Consequently, a QCM resonator may be considered as a bank of resonating filters, enabling simultaneous/discrete characterization of the same load over different frequencies, increasing the correlated information achievable through a single sensor implementation [76,77]. A recent work implemented the multiple-overtones analysis technique for a biosensing application, utilizing an impedance analyzer with a frequency sweeping range from 20 Hz to 120 MHz. Thus, allowing for a practical evaluation of a 10 MHz crystal up to its 11th overtone, compared to the 23rd overtone for a 5 MHz crystal. Crystals of both frequencies were tested and found to provide stable results, where corresponding BVD parameters for each harmonic were found through non-linear curve fitting [78]. The setup detection limit for $R_{m}$ variations were within the order 0.3 Ω, in addition to dissipation monitoring values of 0.01 µU (*micro dissipation units*). The authors also demonstrated the increased sensitivity with higher overtones, yet, at the cost of increased losses and decreased quality factor [78]. Collectively, the utilization of conventional (5–10 MHz) QCM resonators at higher overtones is a practically viable solution for applications requiring increased sensitivity levels, when compared to the thinner, more expensive crystals with higher fundamental frequencies.

On the other hand, different impedance-based electronic interfacing systems were developed by researchers to overcome the main shortcomings of conventional lab-based setup. Although their outcomes are not as accurate, such systems still possess the advantages of low-cost and portable size instruments [38,63,72,73,79,80]. These modified systems may be divided into two main categories based on their operating principle. (1) Circuits utilizing voltage-divider based analysis to obtain the BVD model parameters [38,63,80]. (2) Circuits utilizing a similar operating principle to conventional impedance analyzers (i.e., conductance and susceptance spectra) through compact circuitry [72,73,79].

#### 3.1.2. Voltage-Divider Based Impedance Measurements

**Figure 9:**
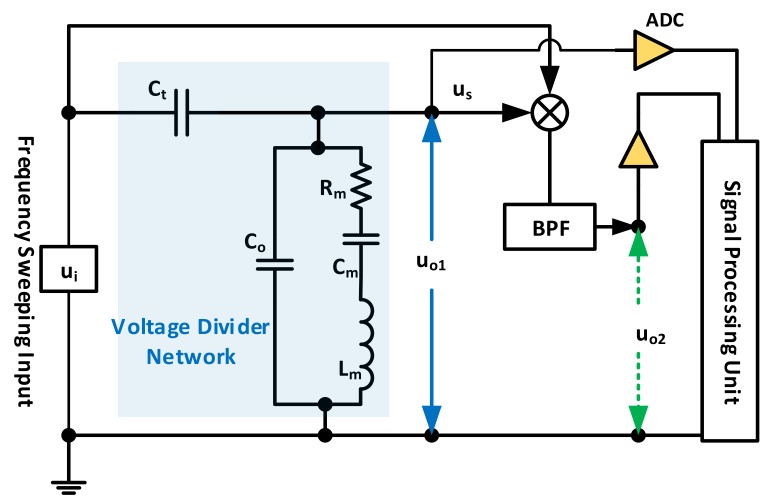
(**Dashed**) Voltage-divider based impedance sweep circuit; (**Overall**) DSB modulated response characterization.

For the first type, circuits complexity may vary based on the required level of accuracy. Figure 9 collectively demonstrates two such systems. The first one, represented inside the voltage divider network box, shows the QCM connected in series to a passive element to establish voltage division. A capacitor is deemed more practical by the authors of [4] due to the ability to select its value accurately as it exhibits a close-to-ideal behavior. The transfer function $\left|u_{o1}/u_{i}\right|$ is obtained as in Equation (9), where $C_{t}$ is the series connected capacitor to the network.

(9) $$\left|\frac{u_{o1}}{u_{i}}\right|\;=\frac{\sqrt{R_{m}^{2}+{\left(\omega L_{m}-\frac{1}{\omega C_{m}}\right)}^{2}}}{\sqrt{{\left(R_{m}+\left(\frac{R_{m}C_{o}}{C_{t}}\right)\right)}^{2}+{\left(\omega L_{m}-\frac{1}{\omega C_{m}}+\frac{\omega L_{m}C_{o}}{C_{t}}-\frac{C_{o}}{\omega C_{t}C_{m}}-\frac{1}{\omega C_{t}}\right)}^{2}}\;}$$

By sweeping f around the resonance region, experimental results of $\left|u_{o1}/u_{i}\right|$ were obtained. From here, BVD parameters may be estimated through non-linear (e.g., least-squares) curve fitting, with adequate initial and boundary conditions based on the expected operating ranges.

An enhancement to this technique is presented in [80] and is represented by Figure 9 through the outer $u_{o2}/u_{i}$ loop. Namely, the interrogating signal is modulated by a low-frequency Double Sideband Amplitude Modulated (*DSB-AM*) signal of frequency $f_{m}$. The resulting input sweeps the resonance region within an additional bandwidth of 2$f_{m}.$ Applying this input to the voltage divider network and mixing it with the QCM output with appropriate filtering only passes signals with double the low modulating frequency 2$f_{m}$. This signal carries information about both real and imaginary QCM impedance parts and thus a similar non-linear fitting technique can be used to extract the sensor’s equivalent circuit parameters. The mathematical derivation is given with respect to LEM in [80] and is simplified further in [63]. Major advantages of this method are its noise immunity since its output consists of two coherent signals, in addition to its variable-controlled sensitivity that is determined via the modulating frequency selection. This property is mainly advantageous in case of heavy-loaded QCM resonators.

**Figure 10:**
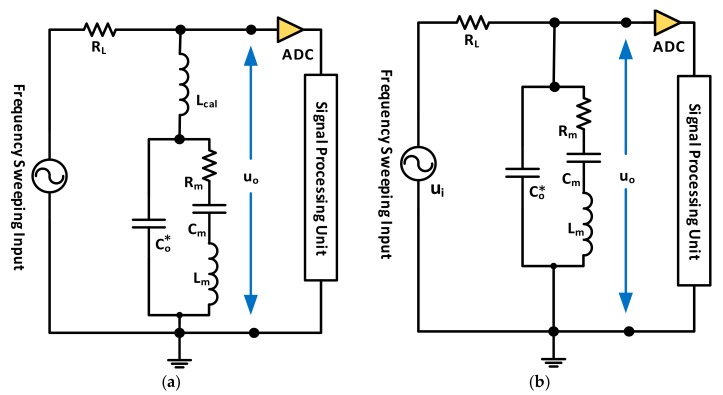
BVD parameters estimation system from [38] for (**a**) $C_{o}$; (**b**) $R_{m}$.

Another recent work discussed a similar characterization technique to empirically estimate $R_{m}$ and $C_{o}^{*}$, without requiring the non-linear curve fitting [38], indicating that $L_{m}$ and $C_{m}$ may be estimated through Equation (6). Yet, the utilized $C_{o}$ for this calculation should exclude $C_{ext}$ effect as it does not influence the motional parameters, thus requiring precise $C_{ext}$ calibration/measurement, which is not always straightforward. The basic idea for $C_{o}^{*}$ estimation is to connect a calibration inductor $L_{cal}$ in series to the sensor, so that it resonates with $C_{o}^{*}$ at a frequency that is selected to be far away from the QCM actual resonance, in a region where $C_{o}^{*}$ dominates the sensor’s impedance (i.e., where the motional arm is essentially an open circuit compared to $X_{C_{o}^{*}}).$ The required circuitry for this test is presented in Figure 10a, where a load resistor $R_{L}$ is connected to protect the circuit at resonance. Furthermore, $L_{cal}$ value needs to be precisely identified prior to the actual test as it is essential for accurate characterization. Thus, the inductor is calibrated first through a different *RLC* calibration circuit with precisely known values of R and *C* [38]. Once calibrated and implemented, the network in Figure 10a is passively interrogated through a sweeping signal around the expected $L_{cal}C_{o}^{*}$ resonance for minimum output signal detection, corresponding to $f_{cal}$. From there, $C_{o}^{*}$ is estimated as in Equation (10).

(10) $$C_{o}^{*}=\frac{1}{{\left(2\pi f_{cal}\right)}^{2}L_{cal}}$$

After that, the inductor is disconnected from the circuit and it reduces to that in Figure 10b, where the $R_{L}$ resistor, whose value should also be precisely identified, is used to estimate $R_{m}$. Nevertheless, it is advisable to set $R_{L}$ value around the expected $R_{s}^{T}$ to avoid non-proportional, noisy measurements. Consequently, the network is passively interrogated, in this case with a signal whose frequency sweeps the QCM series resonance region. Once identified as the maximum output $u_{o}$ point, the motional branch is reduced to $R_{m}$. The parallel $C_{o}^{*}$ effect is neglected by the authors in [38] as tests were conducted for unloaded resonators, indicating the estimation accuracy for lightly-loaded resonators only, based on Figure 5 explanation (i.e., for the given scenario, $R_{s}^{T}$ is estimated as 377 Ω rather than 400 Ω without proper $C_{o}^{*}$ compensation). Thus, $R_{m}$ is estimated at this frequency for lightly loaded sensors by means of a simple voltage divider circuit as in Equation (11).

(11) $$u_{o}=\frac{R_{m}}{R_{m}+R_{L}}u_{i}\;\to \;R_{m}=R_{L}\frac{u_{o}}{u_{i}-u_{o}}$$

The circuits in Figure 10 may be implemented on a single board through connecting a parallel switch to the calibration inductor with minimal on-state resistance to short out its effect when $R_{m}$ estimation is performed for enhanced measurement repeatability.

#### 3.1.3. Compact Impedance Analyzers

The second type of impedance-based QCM characterization, introduced at the beginning of this section, utilizes circuits capable of achieving similar operating principle to conventional impedance analyzers through customized and compact electronics. Such experimental setups typically include passive interrogation of QCM sensors around resonance, generating output signals that can be analyzed through computer-based algorithms and eventually displayed to users. Thus, avoiding the necessity of purchasing expensive and bulky impedance analyzers.

**Figure 11:**
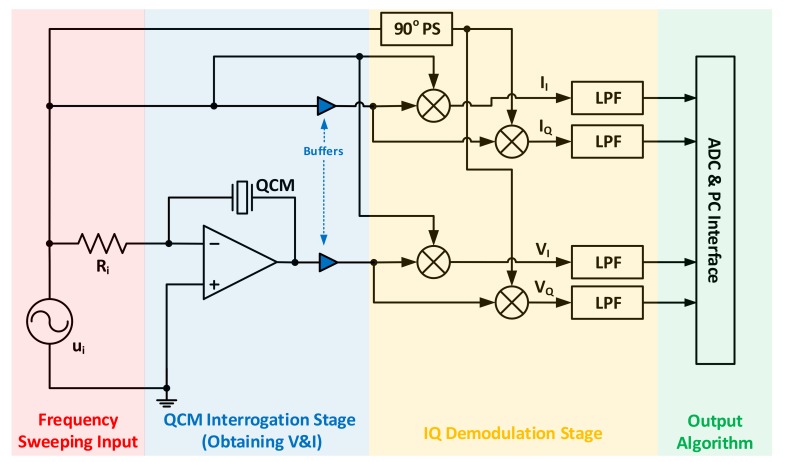
Compact impedance-based characterization system from [73], supporting multiplexing operation.

The work presented in [73] represents such compact system as shown in Figure 11, where the components are carefully selected to exhibit minimum noise/distortion, with sufficient operating bandwidth.

The frequency sweeping input interrogates the QCM, placed within the feedback path of the amplifier. In this configuration, the amplifier converts the interrogating input voltage into a current, which is forced through the QCM. The amplifier output follows the resonator’s voltage drop, where this signal is buffered afterwards and mixed with the interrogating signal and its 90° shifted version to produce in-phase and quadrature-phase (IQ) outputs, representative of the QCM’s voltage. Similar components are produced for the resonator’s forced current [73]. The four signals are fed to LPFs with appropriate cutoff frequencies for demodulation, passing only the relevant components representative of the crystal’s voltage drop |V|∠θ and current |I|∠ϕ through $I_{i},\;I_{q}$ and $V_{i},\;V_{q}$. Eventually, the complex impedance for each interrogating frequency is obtained, as in Equation (12).

(12) $$Z=\frac{\left|V\right|∠\theta \;}{\left|I\right|∠\phi }$$

The authors tested their system on polymer-coated 5-MHz QCM sensors, detecting a maximum conductance of 21.57 mS, compared to 28.05 mS for the conventional HP 4192A LF impedance analyzer at similar frequencies (~100 Hz *of MSRF deviation is also observed)*, indicating appropriate functionality with further requirement of setup optimization. Consequently, the authors demonstrated the possibility of using their developed system in measuring simultaneous responses from different QCM sensors through a multiplexing network that alternates their connection to the system in a synchronized fashion to the programming algorithm [73].

**Figure 12:**
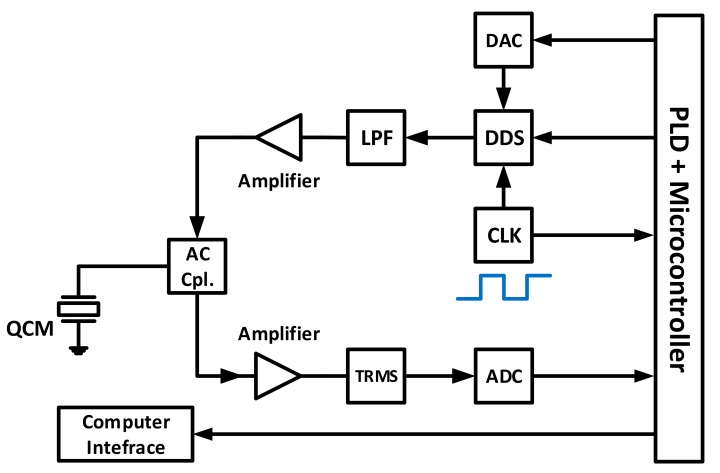
Rapid impedance measurement compact system from [72], based on DDS and PLD controller.

Another compact, rapid QCM impedance scanning system was introduced in [72], with a hand-held size electronic board that is able to scan a 20 kHz bandwidth centered around the resonance frequency at a resolution of 0.2 Hz in 200 ms. This time decreases significantly for lower resolutions, narrower bandwidths. Thus, achieving the objective of a cost-effective platform, where most acquisition tasks are performed at the software level to minimize the hardware interference for any required modification. The system block diagram is shown in Figure 12, where the Programmable Logic Device (*PLD*) controls and regulates its operation. An 80-MHz clock signal is used to drive both the PLD and the Direct Digital Synthesizer (*DDS*), which produces sinusoidal outputs with frequencies up to 15 MHz and amplitudes up to 8.33 $V_{rms}$, controlled through the Digital to Analog Converter (*DAC*) block. The frequency output is filtered for noise reduction and amplified prior to being fed to the QCM resonator through AC-Coupled network. The sensor response is fed back to the network through the RMS to DC converter block (*TRMS*) and eventually fed back to the PLD.

Fast non-linear fitting algorithms are also employed by this system to determine unknown characterization coefficients. Subsequently, the system also allows for QCM characterization at different resonance harmonics [72].

Another system, based on a similar topology and utilizing impedance-matching networks for real-time QCM characterization, has been recently developed and demonstrated high levels of real-time, accurate measurements, suitable for in-situ applications [81]. Applications of compact, impedance-based QCM analysis extend to characterize rapid resonance frequency and dissipation factor (D) variations through expansive frequency range adaptation, achieving integrated low cost instruments with high reported sensitivities, as in [79].

### 3.2. Electronic Oscillators Based Measurement Systems

Electronic oscillating circuits are extensively studied in literature for QCM measurements under several operating conditions. Compared to conventional impedance networks, the sensor is not passively interrogated; instead, it acts as the frequency determining component within the oscillating circuit. Thus, tightly-controlled designs need to minimize interference between QCM and other circuit components for accurate results interpretations [39,63,82]. In this subsection, the principle of operation and design considerations of QCM based electronic oscillators are first briefly introduced, followed by evaluating different existing, widely used circuits in literature.

A linear electronic oscillator is composed of a loop including an amplifier with gain α and a feedback frequency-selective network with a transfer function *β*. The latter acts as a band-pass (*LC*) filter in the case of crystal oscillators, only passing signals within its resonance frequency’s neighborhood, depending on the system’s quality factor-*Q*. On the other hand, the amplifier is designed to restore the decayed amplitude caused by the damping of the filter components. The required amplifier gain for oscillation sustainability is found to be proportional to $R_{m}$ [63]. That is, operating under heavy-loaded conditions increases the effective motional resistance, leading to damped oscillation amplitudes through the frequency selective feedback network (β), comprised mainly of the QCM. The amplifying network (α) gain must proportionally increase to compensate the loop-gain loss. Some customized-oscillator designs employ Automatic Gain Control (*AGC*) amplifiers [9,83] to continuously adjust the gain, following real-time damping level and indicating $R_{m}$. In contrast, other conventional oscillators depend on their active amplifier nonlinearities and saturation behavior for gain control [84]. In all cases, if the amplifier is unable to adequately compensate for the feedback network losses, the oscillation is lost.

Consequently, the requirement to sustain oscillatory behavior at a fixed amplitude for a closed loop circuit, is to maintain the loop gain equal to 1, in addition to having a total loop phase-shift that is equal to zero or multiples of 360° to avoid destructive interference patterns. These two requirements can be summarized as the “*Barkhausen Criteria*”.

(13a) |αβ|=1

(13b) ∠αβ=n360, n=0,1,2, …

Crystal oscillators are well-developed in literature with a wide range of applications due to their high stability and selectivity compared to other oscillators. The detailed principle of operation for conventional crystal oscillators can be found elsewhere [85]. Specific design considerations have to be taken into account when designing QCM electronic oscillating circuits based on the application and operating conditions, especially for highly-damped mediums. For instance, it is recommended to adopt an oscillator design that grounds one crystal side, preferably the immersed electrode, in case of a single-side immersion in liquid for a better control of $C_{o}^{*}$ [86,87].

Some oscillators are inherently designed to drive the crystal at or near its MSRF (i.e., series resonance mode), depending on the medium damping and $C_{o}^{*}$ compensation. Other circuits are designed to drive the crystal in parallel-mode within the inductive region of Figure 3. The resonator, in this case, acts as a high-quality inductor, depending on its *Q* and the system achieves stable oscillation at a frequency between $f_{s}$ and $f_{p}$. In this case, the resonator is typically combined with other passive components in the feedback loop to sustain the Barkhausen loop-phase criteria and their design values determine the operating frequency. Thus, more system controllability is possible with this operating mode when compared to series (*zero-phase, low impedance mode*), although the latter, for negligible or compensated $C_{o}^{*}$ conditions, is more representative of the resonator’s true *MSRF* when absolute frequency monitoring is required. Furthermore, the feedback/amplifying network passive/active components selection must take their long-term stability into account to maintain minimal interference with QCM characterization (i.e., stability vs. temperature/humidity effects, minimal parasitics and non-idealities) [82,88].

Accordingly, a more complex, yet accurate type of electronic oscillator circuits is developed to lock-in the QCM oscillations to its true MSRF. For this type of circuit, VCOs, or similar types, are employed under a Phase-Locked-Loop PLL setup. The VCO frequency varies around a pre-defined range, with the operating point defined by a feedback signal from QCM network, eventually locking to *MSRF* and satisfying the control loop condition based on adequate designs [89,90,91].

**Figure 13:**
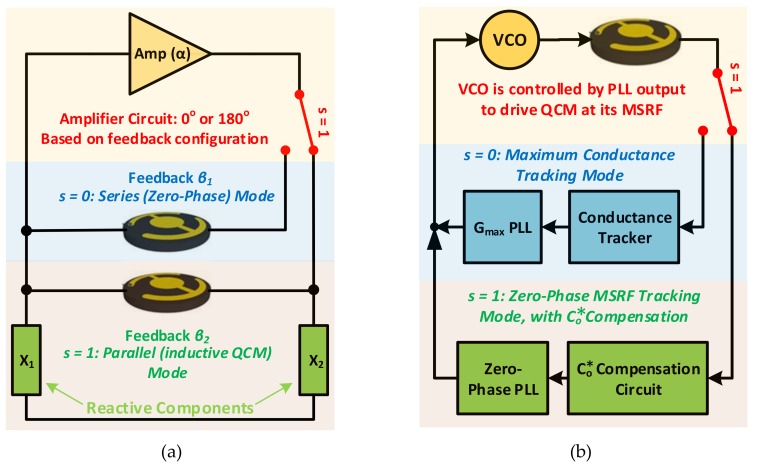
Generic block diagrams of oscillator based QCM electronic interfacing systems: (**a**) self-oscillating modes; (**b**) PLL based MSRF tracking modes.

The following subsections thoroughly analyze and evaluate the different type of oscillator-based QCM electronic interfacing systems. That is: (1) Self-Oscillating QCM Based Oscillators. (2) Controlled (*Lock-In*) Based Oscillators, where VCOs drive the circuit to a frequency satisfying accurate QCM characterization at *MSRF*. Beforehand, generic connections of both options implementations are illustrated in Figure 13, where the switching pattern is presented to showcase the different possibilities for each scenario.

#### 3.2.1. QCM Based Oscillators

Conventional electronic oscillator circuits that are typically used as *LC* tank oscillators were among the first options adopted by QCM researchers [86]. Traditional examples include the Colpitts, Pierce and Miller Oscillators. Taking Colpitts as an example, the QCM operates within the feedback loop as a high-quality inductor $L_{m}^{\prime }$, within the narrow inductive region from Figure 3. The oscillating frequency is thus defined by the resonance frequency between $L_{m}^{\prime }\;$ and feedback load capacitors combination (similar to Figure 13a). Frequency variations are detected through slight changes in $L_{m}^{\prime }$, thus shifting the virtual *LC* resonance point and the oscillation frequency. Yet and although Colpitts inherently possess the advantage of a single grounded crystal’s terminal, it is not considered as a preferred design for highly damped in-liquid applications due to its gain/phase sensitivity to the resonator losses [86]. Some works suggested the connection of additional amplifying stage for improved stability [92]. This option adds to the circuit complexity and risks additional QCM loading without adequate buffering.

Conventional Pierce crystal oscillator has also been traditionally used for QCM applications [86] and general-purpose commercial products (e.g., OpenQCM [93]) under various operating conditions. It provides a good level of stability for lightly-damped application, yet, it possesses similar disadvantages to those of Colpitts under highly-damped conditions, while lacking the grounding of one of its crystal electrodes [86].

In contrast, a double-resonance based oscillator circuit is introduced in [94] and its performance is compared to Colpitts, demonstrating an enhanced oscillating performance under single side or complete distilled water immersion for QCM applications. The proposed circuit oscillation mode is controlled through a variable capacitor, which can be adjusted to lock the circuit oscillation to the crystal’s resonance based on the operating condition. The circuit is extended in [95] through utilizing a variable-capacitance diode for a voltage controlled resonance mode.

##### Miller Oscillator

**Figure 14:**
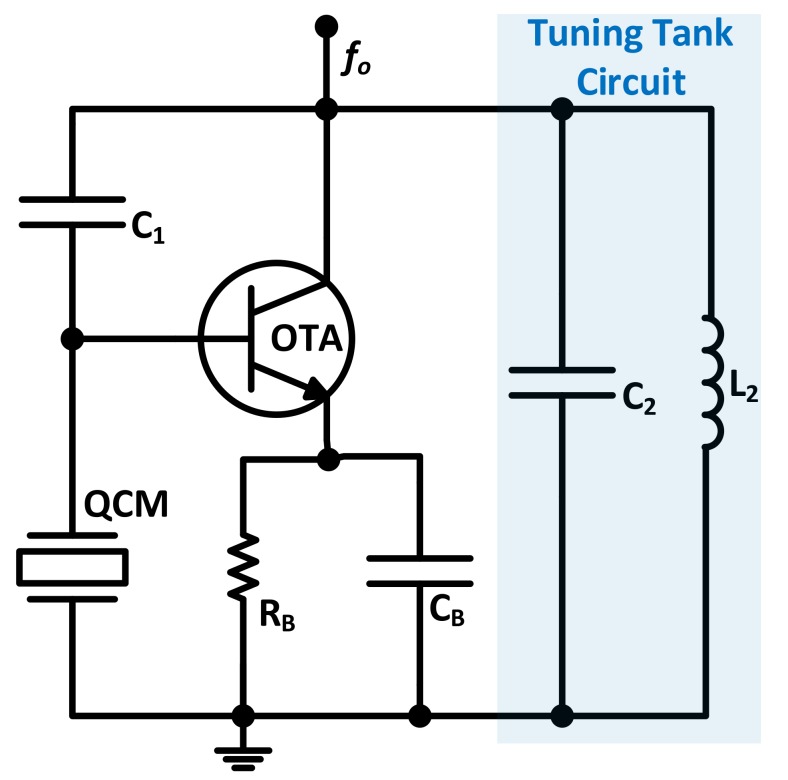
QCM based Miller oscillator implementation, from [97].

The Miller circuit configuration has also caught the attention of some research groups for its functionality under a wide range of operating conditions [96,97,98]. Similarly to Colpitts, several versions of the circuit exist with different simplifications. However, the one that is widely used in literature is based on the utilization of a non-inverting Operational Transconductance Amplifier (*OTA*) as its active device [97] as shown in Figure 14. It is experimentally advised that the resonating frequency associated to the $L_{2}C_{2}$ filter should be selected below the expected QCM lower oscillating frequency limit to minimize the circuit’s frequency dependence on the filter.

An optimization algorithm for optimal QCM oscillators design is presented by Rodríguez-Pardo et al. [99] and is applied to different Miller circuit configurations in [96,97]. The algorithm sets to optimally determine circuit component values/ranges based on the application’s expected operating conditions.

The Miller configuration is advantageous for in-liquid applications (e.g., biological and electrochemical) as one of its crystal electrodes is inherently grounded. The circuit in Figure 14 was implemented in [98] for in-liquid biological sensing application, using a 50 MHz crystal, with satisfactory performance. When compared to the Active Bridge Oscillator (*ABO*), Miller oscillator was also found to maintain an enhanced noise performance with comparable frequency stability levels, in addition to sustaining oscillation under lower *Q* (i.e., sustaining oscillation under higher damping conditions) [100].

##### Commercial QCM200 Oscillator-Based Instrumentation

Developed by Stanford Research Systems (*SRS*), the commercial QCM200 characterization system has been widely used for gas and liquid-phase QCM applications in different fields [101,102,103,104] due to its adopted Automatic Gain Control (*AGC*) based oscillator design [9]. The system mainly consists of an interfacing panel that is connected to an oscillator module. Consequently, the system supports manual $C_{o}^{*}$ compensation (i.e., $C_{o}$ and external apparatus capacitive influence), in addition to real-time $R_{m}$ monitoring. 

**Figure 15:**
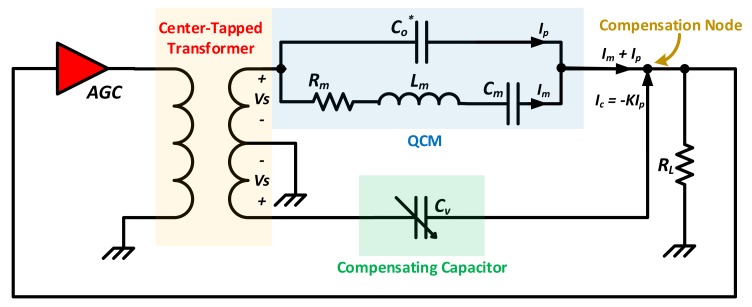
QCM oscillator with parallel capacitance compensation from SRS products.

Figure 15 shows the system’s oscillating circuit design. The uncompensated QCM sensor is connected to the positive output of a center-tapped transformer, driven by an AGC amplifier, whereas a variable compensating capacitor $C_{v}$ is connected to the negative terminal. The same voltage, with opposite polarities, is applied across both $C_{o}^{*}$ and $C_{v}$. The compensating capacitor is connected to the QCM at the compensating node. The total current entering the node is defined as the summation of QCM motional and parallel capacitance currents $I_{m},\;I_{p}$ respectively, in addition to $C_{v}$ current, $I_{c}$, which is related to $-I_{p}$ through their simultaneous voltage. Equation (14) presents the node-entering complex currents.

(14) $$I_{in}=I_{m}+I_{p}\left(1-K\right)$$

A factor K relates both capacitor currents, which is equal to 1 only when $C_{v}=C_{o}^{*}$. Thus, achieving parallel capacitance compensation and reducing the QCM circuit to $R_{m}$ only at *MSRF*. Total compensation is identified as the point of minimum AGC gain for a given operating condition [9]. Once $C_{o}^{*}$ is compensated, the circuit reduces to a simple voltage divider at *MSRF*, consisting of the AGC (*with known gain* $\alpha_{v}$), a load resistor $R_{L}$ and the unknown $R_{m}$. Thus, $R_{m}$ is estimated as in Equation (15).

(15) $$R_{m}=R_{L}\left(\alpha_{v}-1\right)$$

Given the inherent advantages of the QCM200 system, the manual nature of $C_{o}^{*}$ compensation may be considered as a challenging factor due to its potential calibration/drifting errors. In contrast, other methods are introduced in literature for continuous Automatic Capacitance Compensation (*ACC*). Those are discussed further down in Section 3.2.2.

##### Other QCM Oscillators

Many other crystal oscillator circuits, customized for QCM applications, are presented in literature; for instance, the emitter-coupled oscillator. The basic structure of this circuit is based on cascading two common-emitter transistor amplifiers, requiring adequate buffering. The theoretical output of each stage is an inverted amplified version of its input based on the circuit structure. Thus, covering the loop-phase requirement and forcing the crystal to ideally operate at its zero-phase point, which fairly corresponds to *MSRF* for high *Q* crystals with uncompensated $C_{o}^{*}$. Yet, the QCM zero-phase point is considerably deviated from *MSRF* for highly-loaded crystals (low *Q*). Thus, oscillating at the zero-phase frequency in such case without adequate $C_{o}$ compensation may cause significant practical results misinterpretations that should be taken into account through the computing algorithm. Different versions of the design have been discussed in [63], where a single (*OTA*) is used to replace the conventional dual-transistor circuit to enhance the control and stability of its biasing and gain, with additional improvements to the frequency selectivity of the circuit as well. A recent work introduced a compact-oscillator circuit based on a similar configuration that is adequate for in-liquid operation with embedded amplitude control function [105].

**Figure 16:**
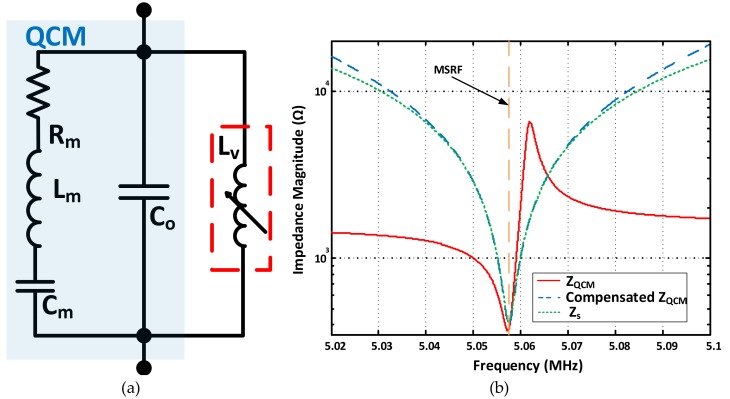
Single-frequency *C_o_* compensation through tuning inductor. (**a**) Circuit diagram; (**b**) Modified impedance response.

Another main category of QCM oscillator circuits is based on bridge oscillators. QCM electronic interfacing systems based on standard, active and lever bridge oscillators are introduced and comprehensively discussed and evaluated in [63]. These circuits are typically designed for the QCM to oscillate at its series (*zero-phase, low-impedance*) frequency. Different methods are also introduced for $C_{o}^{*}$ compensation in bridge oscillators. For instance, through connecting a parallel tuned inductor to the sensor [63]. The compensation circuit diagram is presented in Figure 16a, where the inductor is tuned to produce an equal and opposite reactance to that of $C_{o}^{*}$, exactly at *MSRF*. Taking the same crystal example as before, the parallel capacitance $C_{o}^{*}$ reactance at theoretical *MSRF* is 1.573 kΩ. Then, a parallel inductor value of $L_{v}=\frac{X_{MSRF}}{2\pi f_{MRSF}}=49.51\;\text{µ}H$ is required to achieve complete $C_{o}^{*}\;$ compensation. Figure 16b illustrates the compensation curve, where the compensated QCM collective impedance approaches that of the series motional arm $Z_{m}$ exactly at MSRF. Yet, the compensation inductance *L_v_* calculation is not straightforward, especially when $C_{o}^{*}$ and true MSRF values are not precisely known a priori, thus, practical tuning and calibration may be required for the given operating point. In addition, passive compensation techniques, using $L_{v}$ or $C_{v}$ as in the *QCM200* system, are limited to a single frequency compensation per-tune. That is, the passive component value ($L_{v}$ or $C_{v}$) needs to be manually adjusted, corresponding to each MSRF variation for optimal performance, adding more complexity to the system operation.

Overall, QCM oscillators are widely used for characterization in gas and liquid phase applications, under different damping conditions. Most of their shortcomings can be compensated through appropriate design and control techniques. Their low-cost and compact circuitry, with the wide design variety that fits different applications, makes them a preferred choice for many QCM applications that aim at studying deposited films and liquids properties.

#### 3.2.2. Controlled (Lock-In) Based Oscillators

PLL based characterization systems for QCM resonators maintain the oscillator circuits low-integration cost, while providing more accurate results through automatic *MSRF* tracking. That is, the QCM does not operate as the frequency determining component within a self-oscillating circuit in this case, rather, it is driven by an external oscillator that locks to its resonance frequency. Several systems based on the ‘*lock-in’* concept are introduced in literature and can be divided into two main subcategories as in Figure 13b. (1) *MSRF* tracking through locking to the zero-phase point. (2) *MSRF* tracking through maximum conductance point. The first type requires adequate $C_{o}^{*}$ compensation under highly-damped operating conditions, in order for the controlled oscillator, typically a VCO, to drive the mechanical resonator at its *MSRF*. In contrast, parallel capacitance compensation is unnecessary for lock-in systems that are based on maximum conductance point tracking since this point is independent of $C_{o}^{*}$ and is determined solely through the resonator’s motional arm components [106].

##### Zero-Phase Lock-In Circuits

**Figure 17:**
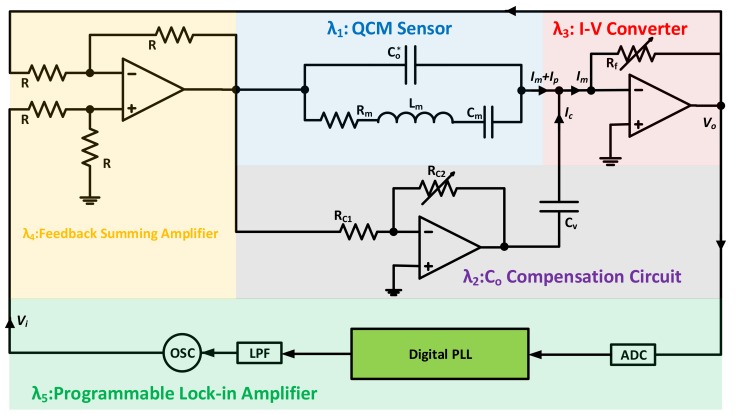
PLL circuit for MSRF tracking with manual Co compensation from [107].

First, PLL systems based on manual capacitance compensation are discussed. An integrated zero-phase lock-in system of this type, presented in [107], is shown in Figure 17. The compensation technique is similar, in essence, to that adopted for QCM200 and Bridge Oscillator. The compensation circuit ($\lambda_{2}$) depends on the adjustable $\xi =R_{c2}/R_{c1}$ ratio, which controls the current injected through $\;C_{v}$. Compensation terms are illustrated through the system’s output/input transfer function in Equation (16a).

(16a) $$\frac{V_{o}\left(s\right)}{V_{i}\left(s\right)}=\frac{\left(s^{2}L_{m}C_{m}+sR_{m}C_{m}+1\right)\;sR_{f}\left(C_{o}-\xi C_{v}\right)-sR_{f}C_{m}}{s^{2}L_{m}C_{m}+s\left(R_{m}-R_{f}\right)C_{m}+1+\left(s^{2}L_{m}C_{m}+sR_{m}C_{m}+1\right)sR_{f}\left(C_{o}^{*}-\xi C_{v}\right)}$$

If ξ is adjusted so that $\xi C_{v}=C_{o}$, then complete compensation is achieved and the system transfer function reduces to Equation (16b), clearly indicating no parasitic capacitance effect on the system response.

(16b) $$\frac{V_{o}\left(s\right)}{V_{i}\left(s\right)}=\frac{sR_{f}C_{m}}{s^{2}L_{m}C_{m}+s\left(R_{m}-R_{f}\right)C_{m}+1}$$

This also has the advantage of effective quality factor improvability through its current-to-voltage ($\lambda_{3}$) and motional current feedback ($\lambda_{4}$) blocks. That is, the adjustable feedback resistor $R_{f}$, controlling the I−V block gain, can be tuned to control the effective *Q* after compensating for $C_{o}^{*}$ as in Equation (17). Yet $R_{f}$ should not be set very close to $R_{m}$ to avoid virtual negative damping and noise accumulation [107].

(17) $$Q_{eff}=\frac{\omega_{MSRF}L_{m}}{R_{m}-R_{f}}$$

Consequently, the resonator is driven by its programmable lock-in amplifier block ($\lambda_{5}$) to its zero-phase frequency (*MSRF*) once $C_{o}^{*}$ is effectively compensated. Practical implementation is also carried out in [107], with satisfactory results. Yet and although the system accurately locks to and tracks its true *MSRF*, it still requires manual tuning of the compensating parameters. The manual tuning process may be inadequate in applications with variable $C_{o}^{*}$ due to medium properties/temperature. High-resolution digitally-controlled potentiometers may be used to replace $R_{c2}$, with automatic adjustment functionality embedded into the system controlling algorithm for simultaneous tracking/compensation action.

Another PLL system based on manual $C_{o}^{*}$ compensation is presented in [76]. The QCM resonator is simultaneously interrogated through two resonance harmonics, namely, the fundamental and third overtone. Two independent PLL oscillators are used for tracking both modes (i.e., each PLL locks-in to its respective harmonic). The developed system is switchless and outputs four signals corresponding to the fundamental and third overtone *MSRFs*, in addition to their motional resistances as in Figure 8. Enhanced sensing capabilities are achieved through the system’s dual-frequency operation, thus increasing the correlated information.

**Figure 18:**
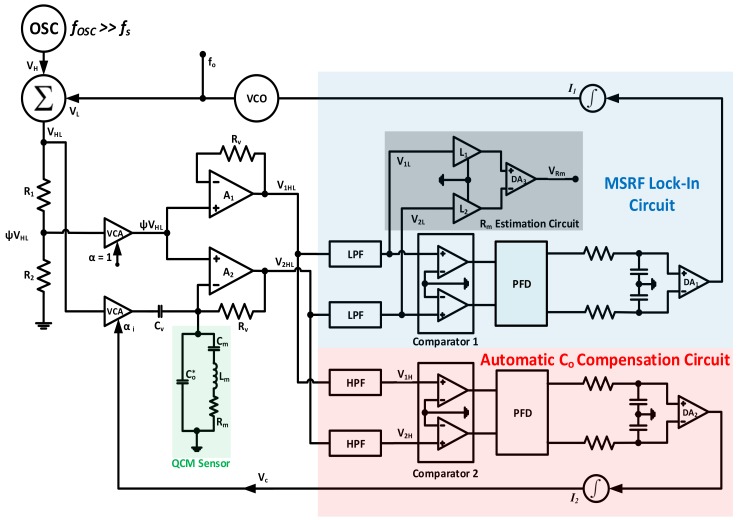
Zero-phase frequency lock-in system with automatic Co compensation, from [88].

On the other hand, the system introduced in [88,108] is a good example of *ACC-based MSRF* tracking interface. The QCM resonator is simultaneously interrogated at two different frequencies: one of them being a fixed high-frequency signal ($f_{H}$), selected ~4 times higher than the QCM fundamental frequency, where the resonator response is mainly dominated by $X_{C_{o}}$. Parallel capacitance compensation PLL is controlled through $f_{H}$ signals. The other interrogating frequency is based on a VCO output that sweeps the resonator’s fundamental resonance region ($f_{L}$) and is responsible for *MSRF* tracking. Both frequencies are added at the circuit input to form the $V_{HL}$ signal for simultaneous interrogation. The complete system’s electronic interface is shown in Figure 18.

The QCM resonator is interrogated by the conditioned $\alpha V_{HL}$ signal and the response is processed through the non-inverting amplifiers $A_{1}$ and $A_{2}$. The amplifiers are selected with high bandwidth to pass the superimposed $f_{L}$ and $f_{H}$ signals without significant attenuation. $A_{1}$ buffers its input as $V_{1HL}=\psi V_{HL}$, where ψ is the voltage-divider ratio $R_{2}/\left(R1+R2\right)$. $V_{2HL}$, on the other hand, is obtained as in Equation (18).

(18) $$V_{2HL}=V_{1HL}\left(1+R_{v}Y_{m}+j\omega R_{v}\left(C_{o}^{*}-C_{c}\right)\right)$$

This signal is considered as the system’s cornerstone, containing the relevant capacitor compensation and *MSRF* tracking information at both frequencies. $Y_{s}=G_{s}+jB_{s}$ is the motional arm admittance and $C_{c}=C_{v}\left(\frac{V_{c}}{\psi -1}\right)$. As stated, $C_{o}^{*}$ compensation is performed at the fixed higher frequency $f_{H}$. Thus, passing $V_{1HL}\;\mathrm{and}\;V_{2HL}$ signals through HPFs with adequate cutoff frequency to filter out $f_{L}$, corresponding to cancelling the $R_{v}Y_{m}$ term from Equation (18) since the motional admittance at $f_{H}$ is essentially zero. Consequently, the filtered $V_{2H}$ and $V_{1H}$ relation and phase difference may be obtained as in Equation (19).

(19a) $$V_{2H}=V_{1H}\left(1+j\omega_{H}R_{v}\left(C_{o}^{*}-C_{c}\right)\right)$$

(19b) $$\phi_{H21}=\tan^{-1}(\omega_{H}R_{v}\left(C_{o}^{*}-C_{c}\right))$$

$C_{o}^{*}$ is fully compensated when $C_{o}^{*}-C_{c}=0$, which is achieved at $\phi_{H21}=0$. The Phase-Frequency Detector (*PFD*) output is proportional to $\phi_{H21}$ and drives a difference amplifier *DA_2_* that controls the integrator $I_{2}$ and its output. That is, when $\phi_{H21}=0$, the integrator outputs a steady DC signal that maintains a constant $V_{c}$ to continuously preserve $C_{o}^{*}$ compensation. From here, the $j\omega R_{v}\left(C_{o}^{*}-C_{c}\right)$ term from Equation (18) is cancelled due to $C_{o}^{*}$ compensation. $V_{1HL}\;and\;V_{2HL}$ are passed through LPFs to cancel $f_{H}$ and the equation reduces as in Equation (20a), with $\phi_{L21}$ relation expressed in Equation (20b).

(20a) $$V_{2L}=V_{1L}\left(1+R_{v}\left(G_{m}+jB_{m}\right)\right)$$

(20b) $$\phi_{L21}={tan}^{-1}\left(\frac{R_{v}B_{m}}{1+R_{v}G_{m}}\right)$$

Similarly, $\phi_{L21}=0$ only when $B_{m}=0$ (i.e., at maximum conductance frequency MSRF). Integrator $I_{1}$ zero input in this case corresponds to a constant DC output, driving the VCO at *MSRF* and achieving simultaneous *ACC* and *MSRF* tracking. In addition, Equation (20a) is reduced at resonance to $V_{2L}=V_{1L}\left(1+\left(R_{v}/R_{m}\right)\right)$. Thus, $V_{1L}$ and $V_{2L}$ signals may be used as in Figure 18 to produce a signal indicative of $R_{m}$ and the sensor damping at *MSRF*.

Similar PLL based systems for QCM characterization are also discussed in [63], including ACC systems similar to the one just described, where capacitance compensation is performed at lower frequencies (e.g., 50 kHz), rather than resonance multiples to avoid amplifiers mismatches at higher frequencies. The sensor response is also highly capacitive for 50 kHz interrogation. Yet, circuit terminology for $f_{L}\;\mathrm{and}\;f_{H}$ are exchanged for the modified circuit.

Another automatic PLL system for MSRF tracking is presented in [91]. The system utilizes a 4-channel DDS for sensor excitation through a numerically controlled oscillator, allowing for simultaneous measurements of up to three different frequencies for enhanced sensitivity through overtones interrogation. Also, the PLL algorithm is mostly executed in the digital domain, thus reducing the required analog electronics (i.e., multipliers and PFDs). Accordingly, the system also has an inherent ACC capability in a similar fashion to that described earlier [91].

Finally, practical implementations of the abovementioned ACC circuits showed good MSRF tracking results. However, slight deviations were observed when compared to results obtained using impedance analyzers due to the ACC circuit components non-idealities. Thus, it is essential to use highly stable components for such circuits [109].

##### Maximum Conductance Lock-In Circuits

**Figure 19:**
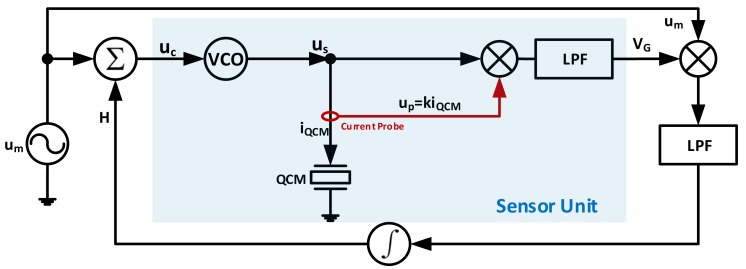
Maximum conductance PLL block diagram from [106].

A different way of looking into the PLL QCM characterization is through maximum conductance frequency tracking. A circuit developed for *MSRF* tracking through this technique is presented in [106]. The instrumentation circuit is composed of a sensing unit to identify the conductance at the interrogation frequency and a control unit to track and lock-to the frequency corresponding to the maximum conductance point. The system is shown in Figure 19, where the VCO output ($u_{s}$) interrogates the sensor through a current probe to get a responsive signal of the sensor current at interrogating frequency ($u_{p}$) as in Equation (21a). The VCO and QCM voltages are then multiplied and passed through a LPF to get a signal $V_{G}$, that is proportional to the QCM admittance phase angle as in Equation (21b). That is, the DC cosine term multiplication simulates obtaining the real admittance part (i.e., conductance). Thus, the conductance signal is maximized at ϕ=0, without a practical need for $C_{o}^{*}$ compensation.

(21a) $$u_{s}\cos\left(\omega t\right)\cdot u_{p}\cos\left(\omega t+\phi \right)=\frac{u_{s}u_{p}}{2}\left[\cos\left(-\phi \right)+\cos\left(2\omega t+\phi \right)\right]$$

(21b) $$V_{G}=\frac{u_{s}u_{p}}{2}\cos\left(-\phi \right)\propto G$$

In order to track the maximum of $V_{G}$, the sensor unit is connected as part of an integrated control circuit, where a synchronous detector composed of a multiplier and another LPF is used and the conductance signal $V_{G}$ is frequency modulated through a low-frequency signal $u_{m}$. The filter output is fed-back to the system through an integrator and added to $u_{m}$, forming the VCO control signal ($u_{c}$) and interrogating the sensor through both carrier and sweeping, modulating, signals.

**Figure 20:**
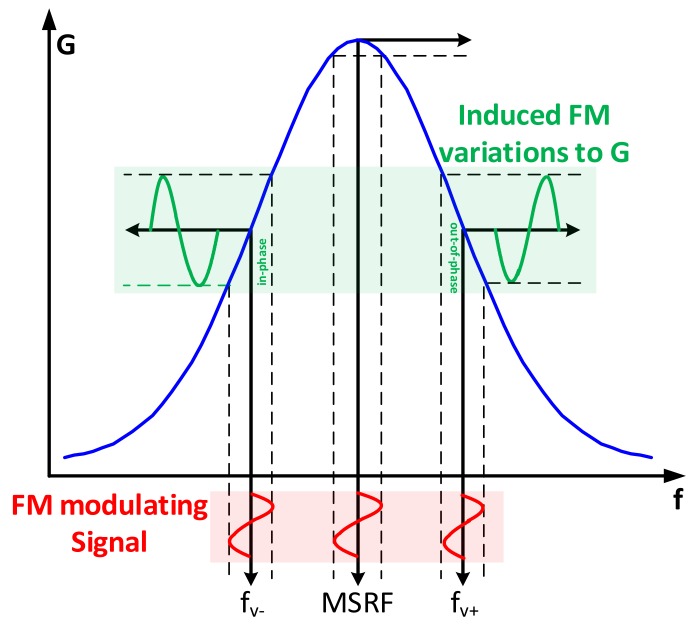
Automatic maximum conductance detection system tuning principle.

The conduction peak detection methodology through modulated $V_{G}$ is depicted in Figure 20. The logic here is that the induced *FM* variations to $V_{G}$ are either in phase or out of phase compared to the modulation signal $u_{m}$ itself, resulting into a non-zero value for the control input *H*. That is, if the VCO output frequency $f_{v}<MSRF$, then the induced FM variations to $V_{G}$ amplitude are in-phase with $u_{m}$ and the opposite is true for $f_{v}>MSRF$. Eventually, the induced variations to $V_{G}$ vanish when the peak conductance point is reached through the controller, indicating a successful lock-in action to *MSRF*.

The selection of appropriate VCO for the application is essential for adequate operation. That is, the conductance peak flatness for low Q systems require a VCO with higher resolutions to avoid false peak detection. For that, crystal type VCO may be used. However, with the drawback of the limited dynamic frequency range [106].

### 3.3. Exponential Decay Based Characterization

Another widespread QCM characterization technique is based on simultaneous measurements of the sensor’s resonance frequency and dissipation factor (quality factor reciprocal, D=1/Q). The technique was first introduced in 1995 [110] and expanded in 1996 through [3,64,111], patented and commercialized into *Q-Sense* instruments later that year [112,113]. The characterization technique is widely termed as ‘*Quartz Crystal Microbalance with Dissipation Monitoring, QCM-D.’* The simultaneous f and D acquirement as two variables, providing independent characterization details for the adlayer’s mass (*f*) and viscoelastic properties—rigidity (*D*), has allowed for a significant expansion to *QCM* applications spectrum, especially in biological fields [66].

#### 3.3.1. Conventional Exponential Decay Technique

**Figure 21:**
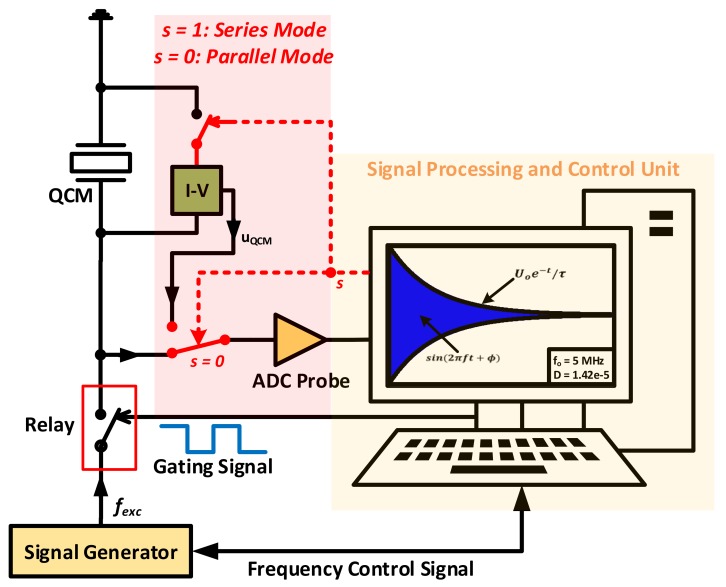
Conventional exponential decay based system for series and parallel resonance detection.

The basic operating principle of this technique relies on passively interrogating the resonator with a signal whose frequency is close to its resonance and disconnecting the source. Thus, causing the excited crystal response to decay exponentially, in a similar manner to a 2nd order *RLC* system as in Equation (22).

(22) $$u\left(t\right)=U_{o}e^{-\frac{t}{\tau }}\;\sin\left(2\pi ft+\phi \right)$$

where $U_{o}$ is the initial oscillations amplitude and A may refer to voltage or current signals, based on the activated series/parallel mode [111] and τ is defined as the decay time constant. Consequently, it is not essential for the excitation frequency $f_{exc}$ to be equal to the sensor’s *MSRF* as it is not known a priori. Yet, the oscillations amplitude $A_{o}$ is increased as $f_{exc}$ approaches resonance, which is advantageous in terms of enhancing measurements resolution [114]. The experimental setup can be modified to excite the sensor at its motional series resonance frequency (i.e., MSRF), or parallel resonance frequency. An equivalent setup for both operating modes is illustrated in Figure 21.

For series mode, the switching parameter *s* is set to 1*,* thus shorting the QCM crystal and its parallel capacitance and effectively reducing the equivalent circuit to its motional branch only. Once the exciting source is disconnected, the decaying crystal’s short circuit current is measured through the current probe (*I-V converter*). The converted signal is passed through an ADC, where the experimental data is digitally processed and numerically fit based on Equation (22) to extract the oscillation frequency f and dissipation factor D, where the dissipation factor is obtained through the fitting parameters as in Equation (23).

(23) $$D=\frac{E_{dissipated}}{2\pi E_{stored}}=\frac{1}{Q}=\frac{1}{\pi f\tau }$$

$E_{dissipated}$ and $E_{stored}$ are the dissipated and stored energies per oscillation, respectively. Consequently, the *MSRF* is estimated as in Equation (24).

(24) $$MSRF\approx \frac{f}{\sqrt{1-\frac{1}{{\left(2Q\right)}^{2}}}}$$

As for the parallel resonance frequency measurement, then the sensor is disconnected from the interrogating source, with s=0 in Figure 21 and left to oscillate freely without shorting its terminals as in the previous case. The sensor’s equivalent circuit thus maintains $C_{o}$ effect and the extracted frequency resembles parallel resonance [111], given that such measurement is essential for some applications (e.g., assessing liquids conductivity) [64,111]. QCM-D based setups are advantageous in terms of their reported accuracy and reduced cost compared to conventional network analysis platforms, while requiring high-quality components for accurate measurements. Generally, QCM-D based analysis are more suitable for lab environments through their relevant products [39].

Accordingly, the introduced concept also allows for sensor characterization at multiple odd harmonics [48], where some improvements were introduced to the original setup in the research group’s later works, where two interrogating signals were simultaneously used, driving the resonator at two different harmonic overtones. Namely, one frequency maintains a similar measurement to that described earlier by measuring f and D at a specific resonance harmonic (*probe mode*); whereas the other frequency signal is used to continuously excite the QCM sensor at another harmonic (*actuator mode*), with variable driving amplitudes to simultaneously influence surface binding reactions and study the effects of sensor’s surface shear oscillations influence on the adsorption kinetics. Appropriate filtering is utilized to separate the responses readouts from both harmonics (i.e., either HPF or LPF depending on the utilized interrogating harmonics) [48]. Accordingly, typical QCM-D systems are reported to be operational with harmonic overtones up to 70 MHz [41,78].

#### 3.3.2. Contactless QCM-D Based Systems

The repetitive excitation/decay nature of this technique attracted researchers’ attention to expand its application into wireless-contactless systems, based on electrical transformers action. Contactless interrogation may be useful for many in-situ applications under different operating conditions in gas or liquid phase, especially when electrical wirings are not allowed (e.g., sealed environments, food packages quality monitoring) [114]. Subsequently, two main wireless *QCM-D* interrogation categories are identified from literature, namely, through operating the crystal either with or without its metal electrodes.

**Figure 22:**
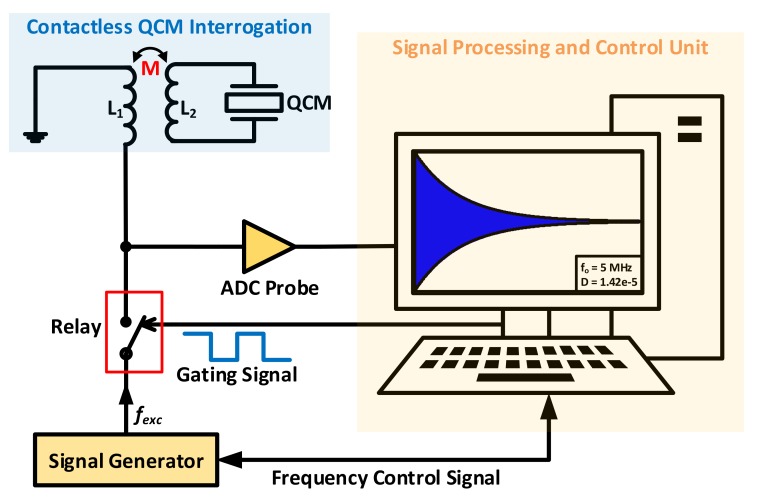
Contactless QCM-D based characterization system from [114].

Recent works proposed contactless based QCM-D system, maintaining the metallic electrodes [114,115,116]. The experimental setup from [114] is depicted in Figure 22, where the excitation signal $f_{exc}$ is connected to the primary coil ($L_{1}$) of an isolated transformer. The QCM resonator, on the other hand, is connected across the secondary coil ($L_{2}$). Once $f_{exc}$ is disconnected, the resonator engages in a similar exponential decay to that explained earlier, which is transferred to the read-out circuit and digital interface through the transformer coils.

The system response was found to be independent, to the first degree, of the interrogating distance between the transformer windings and stable operation was observed experimentally with up to 20 mm separating the coils. That is, increasing the distance mainly reduces the oscillation amplitude, whereas the critical information is independently carried through frequency.

It is important to note that connecting the QCM resonator across a non-ideal inductor, even with a small value, influences the detected frequencies. Yet, the authors of [114] presented an approximation-based closed form solution for estimating the system’s complex resonance frequencies, representing both the mechanical crystal frequency $\left(\omega_{mu}\right)$ and the electrical resonance $\left(\omega_{eu}\right)$ arising from the parallel $L_{2}C_{o}$ pair. Such approximation is held accurate only for lightly damped/undamped resonators, as it assumes decoupled electrical ($L_{2}C_{o}$) and mechanical (QCM) systems, unlike the real case in Figure 22, since the resonator is physically connected to the transformer’s secondary winding. Both systems parameters are obtained through solving a 4th order characteristic equation for the approximated undamped system as in Equation (25). The interconnected influence of both mechanical/electrical systems is evident from the solutions obtained in Equation (26).

(25) $$D\left(s\right)\approx L_{m}C_{m}L_{2}C_{o}\left(s^{4}+\left(\frac{1}{L_{m}C_{m}}+\frac{1}{L_{2}C_{o}}+\frac{1}{L_{m}C_{o}}\right)s^{2}+\frac{1}{L_{m}C_{m}L_{2}C_{o}}\right)$$

(26a) $$\omega_{mu}={\left[\frac{\left(\omega_{MSRF}^{2}+\omega_{eo}^{2}+\delta \omega^{2}\right)+\sqrt{\left(\omega_{MSRF}^{2}+\omega_{eo}^{2}+\delta \omega^{2}\right)-4\omega_{MSRF}^{2}\omega_{eo}^{2}}\;}{2}\right]}^{1/2}$$

(26b) $$\omega_{eu}={\left[\frac{\left(\omega_{MSRF}^{2}+\omega_{eo}^{2}+\delta \omega^{2}\right)-\sqrt{\left(\omega_{MSRF}^{2}+\omega_{eo}^{2}+\delta \omega^{2}\right)-4\omega_{MSRF}^{2}\omega_{eo}^{2}}\;}{2}\right]}^{1/2}$$

where $\omega_{eo}={\left(L_{2}C_{o}\right)}^{-1/2}$ and $\delta \omega ={\left(L_{m}C_{o}\right)}^{-1/2}$. Accordingly, the valid assumption that $X_{L2}\ll X_{Co}$ at resonance, given that $L_{2}$ has a typical value within few µH, reduces the expressions in Equation (26) through Taylor series approximation as in Equation (27), where the terms $L_{2}/L_{m}$ are also practically negligible since $L_{m}$ is typically larger than $L_{2}$ within three orders of magnitude.

(27a) $$\omega_{mu}\approx \omega_{MSRF}\;\left(1-\frac{1}{2}\frac{L_{2}}{L_{m}}\right)$$

(27b) $$\omega_{eu}\approx \omega_{e_{o}}\;\left(1+\frac{1}{2}\frac{L_{2}}{L_{m}}\right)$$

Eventually, the practical readout frequency may thus be approximated to $\omega_{MSRF}$, based on the given assumptions. These approximations validity is verified and compared through simulations and found to be in good agreement under the undamped assumption. Practical validation was also presented through utilizing the contactless setup for measuring resonance frequency in [114] and previously in [115,116] using similar experimental setups, in addition to quality factor tracking based on autocorrelation techniques. Collectively, the presented works in [114,115,116] successfully demonstrate contactless operation of a QCM-D system and may be expanded to include further enhancements for robust operation under different conditions, especially given the setup contactless advantages compared to others reported in literature. However, it should be emphasized that its given predictions are based on undamped/lightly-damped operating conditions (i.e., gas-phase, dry applications). In contrast, such approximations may cause significant variations in practical results vs. theoretical predictions for highly-damped in-liquid applications, when *MSRF* tracking is required.

On the other hand, electrodeless QCM-D based systems are also recently reported in the literature. The electrodeless crystal configuration significantly boosts its sensitivity and limits of detection [42], in addition to overcoming the metallic electrode’s biotoxicity to certain bioactive coating films by directly applying them to the bare crystal. QCM-D is advantageous for such films to assess their real-time viscoelastic changes through dissipation monitoring. Several electrodeless QCM characterization systems are reported in the literature [42,43,44,45,46]. The work presented in [42] utilizes a bare-crystal separately placed in a test chamber. An interrogating signal is radiated through *RF* coil to the crystal. Once the source is turned off, the interrogated signal follows the exponential decay response, which is wirelessly detected by the receiving *RF* coil. The received signal is conditioned, amplified and processed for f and D extraction. For the reported work, an interrogation distance of 3 mm was achieved, allowing to place the bare crystal in closed environments for characterization [42]. Another inherent advantage for the electrodeless setup is the parallel plates capacitance $C_{o}$ absence and thus minimizing the interference with *MSRF* determination. Also, the system is tested under harmonic overtones interrogation conditions, where the measuring sensitivity is tripled at 3rd harmonic, using the same crystal. Thus, enhanced sensitivities may be adequately achieved with non-contact configurations at higher-order harmonics, while providing adequate noise-control [82].

### 3.4. Phase-Shift Based Characterization

Enhancing the experimental QCM sensitivity and limits of detection have always been regarded by researchers as main design challenges, especially for applications requiring high-resolution measurements, where frequency shifts of tiny orders are expected. In contrast, Equation (1) predicts a direct proportionality between resonance frequency and sensitivity. Yet, this relation is not a straightforward, especially when such resonator is implemented within conventional QCM oscillator circuits. That is, the elevated resonance frequency increases the circuit phase-noise and negatively affects its output signal-to-noise ratio (*SNR*) and limits its operation limits [82]. On the other hand, utilizing conventional impedance analyzers is not always feasible due to their high cost and large physical dimensions; whereas QCM-D based systems are also reported to have practical limitations near their limit of detection. Alternatively, the presented characterization technique in this section was shown to overcome the aforementioned practical limitations. The concept of QCM phase-shift based characterization was introduced and patented in the late 1990s [117]. The underlying concept is to interrogate the resonator with a constant, very stable external frequency source and measure the load induced phase-shift at the output. This characterization technique is advantageous compared to conventional oscillators, for instance, in terms of its provided level of stability, sensitivity and resolution, while maintaining a minimal influence of external circuit components. The concept was later expanded by different research groups and experimentally tested under various operating conditions to validate its robustness [17,41,82,118,119].

An elegant mathematical expression linking the coated surface mass density variations $\left(\Delta m_{c}\right)$ to detectable resonator’s phase shift (Δϕ) through the equivalent surface mass density of the contacting liquid ($m_{L})$ is derived and presented in [41,82], where it defines a mathematical base to the phase-mass characterization concept as in Equation (28).

(28) $$\Delta \phi \;\left(rad\right)\approx -\frac{\Delta m_{c}}{m_{L}}$$

**Figure 23:**
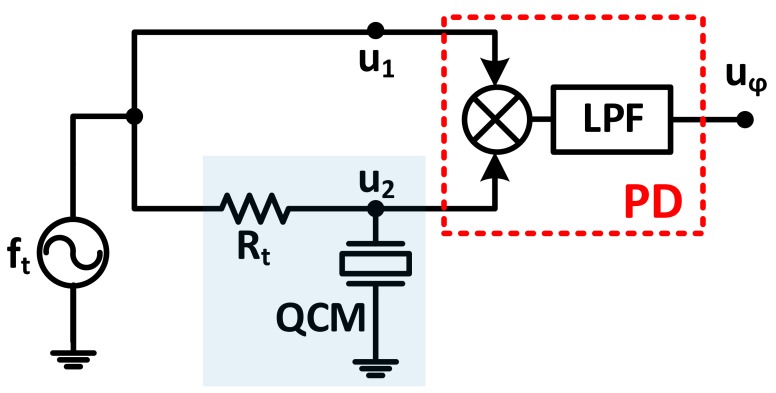
Phase-mass characterization generic block diagram from [82].

Consequently, accurate implementation of Equation (28) requires a stable and adequate electronic interfacing circuit. The setup adopted in [41,82] is based on the generalized diagram depicted in Figure 23. A stable passive interrogation source with a frequency $f_{t}$ (*around resonance*) is connected to the resonator through a voltage divider element $R_{t}$ and the sensor response is mixed with the source signal and passed through a LPF, generating a DC output that is proportional to resonator induced phase-shifts. The mixing-filtering technique is mathematically similar to that explained previously in the maximum conductance lock-in section.

**Figure 24:**
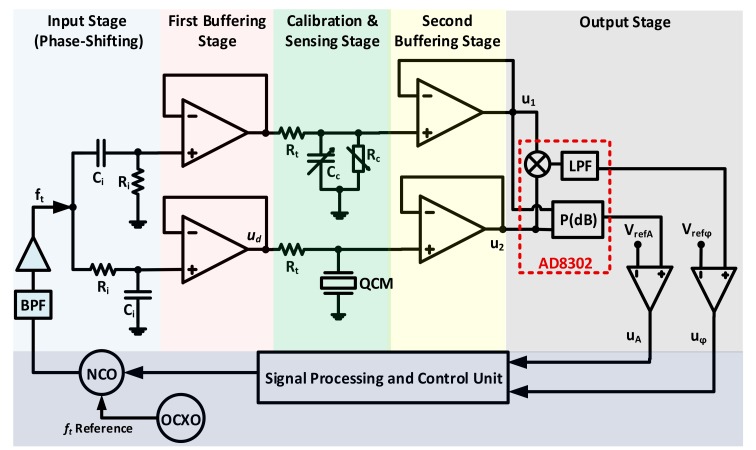
Circuit diagram of the practical phase-mass characterization implementation from [41,82].

The actual circuit used for implementing the phase-mass technique is shown in Figure 24. This experimental setup has been recently patented [120], where different versions of this circuit with slight variations are presented in different works done by the research group [41,82,118,121]. The one presented here is generalized to fully explain the operating principle. The circuit is based on a similar setup to that in Figure 23, consisting of two parallel branches representing the reference interrogating signal $u_{1}$ and the resonator response signal $u_{2}$.

The implemented mixer/LPF block in [82] is based on *AD8302 IC* from Analog Devices, which measures small phase-shifts around 90° between its input signals (i.e., the phase-detector response is centered around 90°). Thus, the input $f_{t}$ is passed through $R_{i}C_{i}$ phase-shifting networks, which are coherently designed around the resonator’s resonance to obtain two orthogonal signals with similar amplitudes. The first buffering stage is included to eliminate loading effects between the input and calibration stages, where the utilized op-amps needs to be carefully selected with a sufficient unity-gain bandwidth (e.g., 100 MHz op-amps were selected in [118] for a 10 MHz crystal). Calibration & sensing stage consists of voltage divider networks at both reference and sensing branches. The reference network components $R_{c}$ and $C_{c}$ resemble the sensor BVD model under resonance (i.e., their values are selected to match $R_{m}$ and $C_{o}$ under the expected operating conditions).

The *IC-AD8302* block is also integrated with a block *‘P(dB)’* that provides an output signal proportional to $u_{1}/u_{2}$ decibel ratio. Additional amplifiers are added at the *IC* output with reference voltages $V_{ref}{}_{\phi }=V_{ref}{}_{A}=900\;\mathrm{mV}$ to center their offset and obtain $u_{\phi }=u_{A}=0$ at 90° phase-shift and unity $u_{1}/u_{2}$ ratio, respectively, providing an easier calibration reference. That is, when *QCM* model is resembled through matching $R_{m}=R_{c}$ and $C_{o}=C_{c}$, the reference and sensing branches outputs are identical and shifted by 90°, thus producing zero $u_{\phi }$ and $u_{A}$ outputs.

Consequent loading of the *QCM* resonator results into varying the output signals, which is correlated to the phase-shift between $u_{2}$ and $u_{1}$, the corresponding shift $\Delta \left(\phi_{u_{2}}-\phi_{u_{1}}\right)$ is mathematically related to interacting mass variations through Equation (29a), where Equation (28) is substituted there to obtain the final approximation as in Equation (29b).

(29a) $$\Delta \left(\phi_{u_{2}}-\phi_{u_{1}}\right)\approx \Delta \phi \left(\frac{R_{t}}{R_{t}+R_{s}^{T}}\right)$$

(29b) $$\Delta \left(\phi_{u_{2}}-\phi_{u_{1}}\right)\approx -\frac{\Delta m_{c}}{m_{L}}\left(\frac{R_{t}}{R_{t}+R_{s}^{T}}\right)$$

From Equation (29), one can notice that setting $R_{t}\gg R_{m}$ further simplifies the equation by approximating its resistive part to 1. Yet, it is not advised for the ratio to exceed $R_{t}=10R_{liq}$ in order to maintain adequate output resolution for the voltage divider network [82]. Accordingly, the signal processing and control unit, implemented through FPGA based system in [82], is used to adjust the numeric control oscillator (*NCO*) output $f_{t}$ around resonance following $u_{\phi }$ and $u_{A}$ feedback, when required. *NCO*’s output is filtered and amplified to maintain a noise-free sinusoidal input. The reference frequency signal is set through an oven controlled crystal oscillator (*OCXO*), with very stable frequency output and minimal phase noise to increase the system level of detection.

The presented setup has been experimentally validated for an immunosensor application in [41] and compared to classical frequency-mass characterization, using a balanced-bridge oscillator, in terms of sensitivity [118]. For a 10 MHz QCM crystal, the phase-mass sensitivity was found to experimentally be 3 times better than that achieved through the bridge oscillator circuit, projecting a much higher expected sensitivity improvement when crystals with higher fundamental frequencies are used, based on the phase-noise problem associated with high-frequency oscillator circuits, as discussed above. The setup has also been tested with HFF-QCM resonators in [119] for immunosensor applications, where 50 MHz and 100 MHz crystals are used. Higher sensitivity was reported through the 100 MHz crystal, compared to 50 MHz and conventional 10 MHz crystals, with $0.14\;\mu g\cdot L^{-1},\;0.23\;\mu g\cdot L^{-1}\;\mathrm{and}\;4.00\;\mu g\cdot L^{-1}$ limits of detection, respectively. Similar experimental setup was also successfully used to characterize Love-wave immunosensors in [122], indicating its adaptability and robustness for various applications.

## 4. High Temperature Applications Design Considerations

### 4.1. High-Temperature QCM Based Systems

AT-cut quartz are widely employed for manufacturing QCM sensors due to their extremely good mechanical and temperature stability within 10–50 °C range as discussed earlier, given that the majority of QCM applications operate within this range (i.e., around the zero-temperature coefficient spectrum). However, other applications require operating the microbalance sensor at elevated temperatures, such as assessing the thermal stability of viscous fluids [10], in addition to high temperature controlled thin-film Atomic Layer Deposition (*ALD*) [11,56].

Consequently, the utilization of AT-cut quartz crystals at high temperatures causes significant frequency drifts influenced by the increased operating temperature [10]. Several compensation techniques were introduced over time to separate the temperature-induced frequency variations from those caused by actual sensor loading. For instance, the use of dual-resonator setup is experimentally verified in [11,15] through exposing two identical resonators to the same temperature conditions, while only exposing one of them to the actual testing environment and physically isolating the other, reference resonator, to limit its detected frequency shifts to temperature variations and prevent film growth on its surface. Thus, establishing a temperature-frequency baseline. The subtraction of both resonators frequency output signals results in a compensated signal that, ideally, represents the surface interactions of the non-isolated resonator. The presented methodology proved an experimentally adequate operation up to 565 °C in [15] and 500 °C in [11].

In contrast, the use of mathematical models to compensate the temperature-frequency drifts is discussed in [11] and compared to the dual-resonator technique. That is, the baseline set earlier through a reference oscillator is set mathematically instead through a polynomial equation, typically of 3rd order, of the form indicated in Equation (30).

(30) $$\Delta F_{T}=a_{3}T^{3}+a_{2}T^{2}+a_{1}T+a_{0}$$

The constants $a_{0}-a_{3}$ are temperature coefficients that are dependent on the crystal cut and properties [10], T is the operating temperature and $\Delta F_{T}$ is the modeled temperature-induced frequency variation. Comparatively good results to those obtained by the dual-resonators system are produced through the model based technique in [11]. Clearly, utilizing the latter technique is advantageous in terms of minimizing the experimental system size and cost by using one resonator only, in addition to overcoming the complexities associated to the reference oscillator isolation. Model-based compensating techniques are also employed for liquid-phase in [123] for determining the state-of-charge of lead acid batteries, while neutralizing electrolyte temperature variations during charging/discharging process.

The aforementioned compensation techniques are meant for adjusting frequency measurements, without addressing the implicit variations in terms of the resonator’s quality factor and BVD parameters, proportional to temperature, from an electrical point of view. Practically, *Q* is found to deteriorate with increasing temperature, such variations are negligible under normal operating conditions within the range of 30–160 °C as reported in [124] and supported by the results presented in [10,56]. Yet, extending the operating temperature further beyond 300 °C and near the reported limit point of 565 °C significantly deteriorates Q and increases $R_{m}$ (i.e., imposing similar experimental effect to that experienced through highly-viscous loading) and necessitating similar experimental considerations for the electronics interfacing circuits design under such harsh operating conditions, such as adequate $C_{o}$ compensation for oscillator circuits.

Eventually, quartz crystal use as piezoelectric resonators is only limited to their phase-transition, Curie-temperature of 573 °C, taking into account that operating QCM resonators near that limit is not recommended given their exponentially increasing temperature coefficient [53]. That is, other piezoelectric crystals with similar sensing properties and higher phase-transition temperatures are more adequate for such applications, maintaining higher mechanical stability and enhanced performance.

### 4.2. High-Temperature Quartz-Alternatives Crystals

Several piezoelectric crystals exhibiting high phase-transition temperatures are used for microbalance applications, mainly, the $GaPO_{4}$ (*GCM*) and more recently, the *langasite (LCM) crystals* [59,125,126]. GCM sensors exhibit irreversible phase-transition around 920 °C, compared to ~1500 °C for LCM resonators [59]. Yet, it is reported that GCM crystals operating above 750 °C exhibit significant *Q* reductions [127], compared to ~1000 °C for LCM sensors [59], supporting the previous recommendation of not operating crystal close to their piezoelectric limits. Extensive practical comparison is performed between GCM and conventional QCM sensors in [53,54,56], demonstrating the former superiority for high-temperature applications. Namely, the GCM motional resistance increments with increasing temperature is significantly less than that of QCM sensors, compared to sharper relative degradations at higher temperatures above 400 °C in terms of GCM quality factor as reported in [56], while maintaining relatively high absolute *Q* values.

In contrast, the Pt-electrodes used for commercial GCM sensors inherently require an adhesion layer that firmly attaches it to the crystal. Several materials have been investigated in literature for this purpose, including tantalum (*Ta*), zirconium (*Zr*) and titanium (*Ti*) [128]. Commercial GCM sensors typically utilize a *Ti* layer, however; titanium diffusion through platinum at temperatures ~600 °C for applications involving oxide-rich environments can form oxide precipitates that affect the adhesion and thus the stability of the electrodes and the overall frequency response as a result. Thus, it is advised to verify the electrodes/adhesion compatibility with the operating environment to avoid any hard-to-detect results misinterpretations that may arise from such problems.

Commercial GCM sensors may be customized to the application requirements by setting its zero-coefficient temperature ($T_{o})$ around the expected operated range, up to 650°, as per the main GCM sensor supplier, *PiezoCryst* [129]. That is, adjusting Equation (30) as follows.

(31) $$\Delta F_{T}\prime =\Delta F_{T}(T_{o})[a_{3}\left(T-T_{o}{)}^{3}+a_{2}{\left(T-T_{o}\right)}^{2}+a_{1}\left(T-T_{o}\right)+a_{0}\right]$$

Yet, associated quality factor degradations around higher operating temperatures, away from the given $T_{o}$ limit for wide-temperature range applications, should be closely monitored when designing the electronic interface due to the aforementioned reasons, mainly, ensuring simultaneous mathematical and practical compensation of any temperature-induced effect.

Consequently, high-temperature microbalance applications, especially with in-situ measurements, require customized sensor holders that are capable of withstanding the harsh operating environment while adequately connecting the sensor to the rest of the readout circuit that is usually kept around room-stable temperature. Non-reactive Gold or Platinum wires are typically used to extend electrical connections as in [57,59,130]. An interesting option for applications requiring customized holder designs, is based on the ‘*pyrophyllite*’ material. This mineral, in its natural state, is easily machined to the required design shape through conventional drilling tools or Computer Numerical Control (*CNC*) machines, before being fired to elevated temperatures  (800 –1300 °C). The fired holder may then continuously operate at high temperatures within the given range. Pyrophyllite based holders have been successfully implemented in several high-temperature GCM applications [8,130].

## 5. Electronic Interfacing Systems Comparison

The discussed characterization techniques may be compared as follows, where Table 1 summarizes the practical advantages and limitations of each discussed electronic interface. Overall, conventional impedance analyzers are superior in terms of their accuracy since the sensor response is interrogated in isolation of external components that may negatively affect its output, in addition to its ability to characterize the sensor at different odd harmonic overtones. Yet, such superiority is conditional in literature to other characterization alternatives such as compact impedance-based systems and oscillator circuits. Based on the operating environment and the sensor damping, parallel capacitance compensation may be pivotal for obtaining accurate results for QCM oscillator circuits, where some oscillator-based PLL systems are designed to automatically compensate for $C_{o}^{*}$ and track the motional resonance frequency. Exponential decay techniques are also considered powerful and adequate in many applications due to their simultaneous f and D measuring capability at multiple overtones and have been recently expanded to include wireless sensor interrogation, which is significant for some in-situ sensing applications. On the other hand, phase-mass characterization is particularly advantageous for high-sensitivity applications when the use of high-frequency oscillating circuits is inadequate due to their amplified phase-noise and reduced SNR. This relatively new characterization technique also supports working with multisensory arrays for extended analysis spectrum.

The special design considerations/compensations discussed for high-temperature applications should also be taken into account when selecting the appropriate characterization technique. For applications involving customized sensor connections, the wiring material/lengths should be kept to a minimum to minimize $C_{ext}$ and maintain it within the compensation range of the selected setup. Finally, microbalance sensors must be handled with extreme care due to their inherently delicate structure to avoid breakage, especially when using high-temperature microbalance sensors as they are much more expensive than conventional QCMs.

## 6. Conclusions

This work presented a comprehensive review covering microbalance sensing applications based on different types of crystals, mainly QCM and GCM. The specific scope of this work was focused on reviewing the existing QCM electronic interfacing circuits and sensor characterization techniques, while identifying the advantages and disadvantages of each in terms of complexity, cost, size and reliability under various operating conditions. The reviewed systems were based on impedance analyzers, electronic oscillators including PLL-based circuits, exponential decay (QCM-D) technique, as well as the emerging phase-mass characterization. Special design considerations for high-temperature applications are also discussed in this work with appropriate compensation techniques and alternative crystals that can operate normally well beyond the quartz Curie temperature limit. Finally, the various covered systems and operating conditions by this work provide the reader with an insightful overview of the existing systems to facilitate her choice for the intended application.

## Figures and Tables

**Table 1:** QCM electronic characterization systems qualitative comparison.

Characterization Technique			Advantages	Limitations
Impedance Analysis	Conventional Impedance Analyzers		Highest accuracy. Supports complete sensor characterization and harmonic overtones analysis. Isolated QCM measurements. Adequate for all mediums, provided that appropriate resolution is supported.	Highest setup cost. Large physical size. Some models require time consuming processing/data fitting for accurate parameters extraction.
	Compact Circuitry	VD Based	Significantly cheaper than conventional analyzers with more compact sizing. Provides essential characterization parameters for different applications (e.g., BVD parameters from [4,80]).	Less accurate compared to conventional analyzers. Sensor is not measured in isolation (part of a circuit). Requires time consuming processing algorithms. Some circuits provide limited characterization [38].
		Compact Analyzers	Maintains most conventional impedance analyzers advantages. Rapid and high-resolution measurements capability [72]. Low cost and high integration capability (handheld size).	Less accurate compared to conventional impedance analyzers.
Oscillator Circuits	QCM Oscillators		Highly integrated low cost circuitry. Good accuracy. Direct frequency measurement. Dissipation monitoring capability for some designs.	Sensor response is influenced by circuit components. Inadequate for overtones study. Requires high components stability. $f_{osc}\neq MSRF$ without $C_{o}^{*}$ compensation in-liquid.
	PLL Based	0-Phase Tracking	Maintains QCM oscillators advantages. Locks in to *MSRF*. Supports simultaneous interrogation by different overtones for extended results. Provides dissipation monitoring capability through signals indicating $R_{m}$ variations.	Requires $C_{o}^{*}$ compensation for *MSRF* tracking. Circuit components influence may affect the tracking accuracy.
		$G_{max}$ Tracking		Requires high-resolution VCO for flat conductance peaks. Circuit components influence may affect the tracking accuracy.
Exponential Decay (QCM-D)	Conventional		Accurately measures series and parallel resonance. Simultaneous f and D tracking capability and overtones excitation.	Fairly expensive due to its high-quality components. Mainly suitable for lab-based applications (i.e., low portability).
	Contactless		Maintains most conventional QCM-D advantages. Extends the applications spectrum to closed volumes (i.e., no QCM wiring). Electrodeless setup increases the measuring sensitivity.	Contactless setup in [114] is limited for lightly-loaded applications. The wireless interrogation distance is still fairly low for various applications.
Phase-Mass Characterization			Achieves higher sensitivity and resolution compared to conventional circuitry. Supports high-frequency crystals characterization with minimized signals noise and interference. Highly integrated setup, supporting sensing arrays.	Current setup provides partial sensor characterization (e.g., limited dissipation monitoring). Limiting the applications spectrum.

## Nomenclature

*ABO*	Active Bridge Oscillator	$L_{m}$	Motional inductance
*ACC*	Automatic Capacitance Compensation	*LPF*	Low-Pass Filter
*ADC*	Analog to Digital Converter	*M*	Mutual Inductance
$A_{e}$	Effective Surface Area	*MSRF*	Motional Series Resonance Frequency
*AGC*	Automatic Gain Control	*N*	Odd harmonic overtone (N = 1,3,...)
*ALD*	Atomic Layer Deposition	*NCO*	Numeric Control Oscillator
*B*	Electrical Susceptance	*OCXO*	Oven-Controlled Crystal Oscillator
*BPF*	Band-Pass Filter	*OTA*	Operational Transconductance Amplifier
*BVD*	Butterworth-Van-Dyke crystal model	*PFD*	Phase-Frequency Detector
$C_{ext}$	External wiring/parasitic capacitance	*PLD*	Programmable Logic Device
$C_{m}$	Motional capacitance	*PLL*	Phase-Locked Loop
$C_{o}$	Parallel electrodes capacitance	*Q*	Crystal’s Quality Factor
$C_{o}^{*}$	Effective total parallel capacitance	*QCM*	Quartz Crystal Microbalance
$c_{P}$	Piezoelectric material elastic constant	$R_{liq}$	Liquid-load damping resistance
*D*	Crystal’s Dissipation Factor	$R_{m}$	Motional resistance
*DAC*	Digital to Analog Converter	$R_{s}^{T}$	Total crystal’s series resistance
*DDS*	Direct Digital Synthesizer	*SNR*	Signal to Noise Ratio
f,ω	Operating frequency, angular form	*t*	time
*FBAR*	Film Bulk Acoustic Resonators	*TSM*	Thickness Shear Mode
$f_{o}$	Unloaded crystal resonance frequency	*u, V*	Voltage
$f_{p}$	Maximum impedance parallel resonance	*VCO*	Voltage Controlled Oscillator
$f_{r}$	Zero-phase series resonance frequency	X	Electrical Reactance
$f_{s}$	Minimum impedance series resonance	*Y*	Electrical Admittance
*G*	Electrical Conductance	*Z*	Electrical Impedance
*GCM*	$GaPO_{4}$ Crystal Microbalance	$\Delta f_{x}$	$f_{o}$ variation, induced by factor x
*h*	Crystal Electrodes separation	τ	Exponential-Decay time constant
*HFF*	High-Fundamental Frequency	ρ	Density
*HPF*	High-Pass Filter	$\epsilon_{p}$	Piezoelectric material permittivity
*I*	Current	$\mu_{c}$	Crystal’s shear modulus
$K_{o}$	Piezoelectric electrochemical constant	η	Viscosity
*LCM*	Langasite Crystal Microbalance	α	Oscillator’s Amplifier gain
*LEM*	Lumped Element Model	β	Oscillator’s Feedback gain

## References

[1] Curie J., Curie P. (1880). An oscillating quartz crystal mass detector. Rendu.

[2] Sauerbrey G. (1959). Verwendung von schwingquarzen zur wägung dünner schichten und zur mikrowägung. Z. Phys..

[3] Rodahl M., Kasemo B. (1996). Frequency and dissipation-factor responses to localized liquid deposits on a QCM electrode. Sens. Actuators B Chem..

[4] Calvo E., Etchenique R., Bartlett P., Singhal K., Santamaria C. (1997). Quartz crystal impedance studies at 10 MHz of viscoelastic liquids and films. Faraday Discuss..

[5] Reed C.E., Kanazawa K.K., Kaufman J.H. (1990). Physical description of a viscoelastically loaded AT-cut quartz resonator. J. Appl. Phys..

[6] Sen K., Ashbolt N. (2011). Environmental Microbiology.

[7] Lu C.S., Lewis O. (1972). Investigation of film-thickness determination by oscillating quartz resonators with large mass load. J. Appl. Phys..

[8] Millichamp J. (2013). Development of a Novel High Temperature Crystal Microbalance In-Situ Sensor for the Study of Electrode Processes in Solid Oxide Fuel Cells. Ph.D. Thesis.

[9] (2011). QCM200 Digital Controller: Operation and Service Manual.

[10] Wang D., Mousavi P., Hauser P.J., Oxenham W., Grant C.S. (2005). Quartz crystal microbalance in elevated temperature viscous liquids: Temperature effect compensation and lubricant degradation monitoring. Colloids Surf. A Physicochem. Eng. Asp..

[11] Rahtu A., Ritala M. (2002). Compensation of temperature effects in quartz crystal microbalance measurements. Appl. Phys. Lett..

[12] Konash P.L., Bastiaans G.J. (1980). Piezoelectric crystals as detectors in liquid chromatography. Anal. Chem..

[13] Nomura T., Okuhara M. (1982). Frequency shifts of piezoelectric quartz crystals immersed in organic liquids. Anal. Chim. Acta..

[14] Keiji Kanazawa K., Gordon J.G. (1985). The oscillation frequency of a quartz resonator in contact with liquid. Anal. Chim. Acta.

[15] Mecea V.M., Carlsson J.O., Bucur R.V. (1996). Extensions of the quartz-crystal-microbalance technique. Sens. Actuators A Phys..

[16] Ishay R.B., Kapp-Barnea Y., Grigoriantz I., Teblum E., Lellouche J.-P. (2015). Real time acoustic profiling of a live cancerous cell monolayer using QCM. Sens. Actuators B Chem..

[17] Wang P., Su J., Gong L., Shen M., Ruths M., Sun H. (2015). Numerical simulation and experimental study of resonance characteristics of QCM-P devices operating in liquid and their application in biological detection. Sens. Actuators B Chem..

[18] Tai H., Bao X., He Y., Du X., Xie G., Jiang Y. (2015). Enhanced formaldehyde-sensing performances of mixed polyethyleneimine-multiwalled carbon nanotubes composite films on quartz crystal microbalance. IEEE Sens. J..

[19] Sharma P., Ghosh A., Tudu B., Sabhapondit S., Baruah B.D., Tamuly P., Bhattacharyya N., Bandyopadhyay R. (2015). Monitoring the fermentation process of black tea using QCM sensor based electronic nose. Sens. Actuators B Chem..

[20] Masdor N.A., Altintas Z., Tothill I.E. (2016). Sensitive detection of Campylobacter jejuni using nanoparticles enhanced QCM sensor. Biosens. Bioelectron..

[21] Subramanian S., Simon S., Gao B., Sjöblom J. (2016). Asphaltene fractionation based on adsorption onto calcium carbonate: Part 1. Characterization of sub-fractions and QCM-D measurements. Colloids Surf. A Physicochem. Eng. Asp..

[22] Jia Y., Yu H., Cai J., Li Z., Dong F. (2017). Explore on the quantitative analysis of specific surface area on sensitivity of polyacrylic acid-based QCM ammonia sensor. Sens. Actuators B Chem..

[23] Yang Y., Zhang W., Guo Z., Zhang Z., Zhu H., Yan R., Zhou L. (2017). Stability enhanced, repeatability improved Parylene-C passivated on QCM sensor for aPTT measurement. Biosens. Bioelectron..

[24] Speller N.C., Siraj N., McCarter K.S., Vaughan S., Warner I.M. (2017). QCM virtual sensor array: Vapor identification and molecular weight approximation. Sens. Actuators B Chem..

[25] Sobiepanek A., Milner-Krawczyk M., Lekka M., Kobiela T. (2017). AFM and QCM-D as tools for the distinction of melanoma cells with a different metastatic potential. Biosens. Bioelectron..

[26] Zajforoushan Moghaddam S., Zhu K., Nyström B., Thormann E. (2017). Thermo-responsive diblock and triblock cationic copolymers at the silica/aqueous interface: A QCM-D and AFM study. J. Colloid Interface Sci..

[27] Lay B., Kandjani A.E., Amin M.H., Kabir K.M.M., Ippolito S.J., Sabri Y.M., Bhargava S.K. (2017). Galvanic replacement of colloidal monolayer crystal on a QCM device for selective detection of mercury vapor. Sens. Actuators B Chem..

[28] Li Q., Gu Y., Wang N.F. (2017). Application of random forest classifier by means of a QCM-based e-nose in the identification of Chinese liquor flavors. IEEE Sens. J..

[29] Speller N.C., Siraj N., Vaughan S., Speller L.N., Warner I.M. (2017). QCM virtual multisensor array for fuel discrimination and detection of gasoline adulteration. Fuel.

[30] Yoo H.Y., Ju M., Bruckenstein S. (2018). Increasing QCM sensitivity based on high molecular weight porous polymer films. Sens. Actuators B Chem..

[31] Chen X., Chen X., Li N., Ding X., Zhao X. (2016). A QCM humidity sensors based on GO/Nafion composite films with enhanced sensitivity. IEEE Sens. J..

[32] Yao Y., Zhang H., Sun J., Ma W., Li L., Li W., Du J. (2017). Novel QCM humidity sensors using stacked black phosphorus nanosheets as sensing film. Sens. Actuators B Chem..

[33] Yao Y., Xue Y. (2015). Impedance analysis of quartz crystal microbalance humidity sensors based on nanodiamond/graphene oxide nanocomposite film. Sens. Actuators B Chem..

[34] Ma W., Tang S., Wei Y., Xie G. (2017). Simple biosensing method to detect DMMP based on QCM transducer and acetylcholine esterase sensitive film. Micro Nano Lett..

[35] Zhu Y., Cheng Z., Xiang Q., Chen X., Xu J. (2017). Synthesis of functionalized mesoporous TiO_2_-SiO_2_ with organic fluoroalcohol as high performance DMMP gas sensor. Sens. Actuators B Chem..

[36] Cooper M.A., Singleton V.T. (2007). A survey of the 2001 to 2005 quartz crystal microbalance biosensor literature: Applications of acoustic physics to the analysis of biomolecular interactions. J. Mol. Recognit..

[37] Becker B., Cooper M.A. (2011). A survey of the 2006–2009 quartz crystal microbalance biosensor literature. J. Mol. Recognit..

[38] Casteleiro-Roca J.L., Calvo-Rolle J.L., Meizoso-Lopez M.C., Piñón-Pazos A., Rodríguez-Gómez B.A. (2014). New approach for the QCM sensors characterization. Sens. Actuators A Phys..

[39] Lucklum R., Eichelbaum F., Janshoff A., Steinem C. (2007). Interface circuits for QCM sensors. Piezoelectric Sensors.

[40] Fernández R., García P., García M., García V.J., Jiménez Y., Arnau A. (2017). Design and validation of a 150 MHz HFFQCM sensor for bio-sensing applications. Sensors.

[41] Montagut Y., García J.V., Jiménez Y., March C., Montoya Á., Arnau A. (2011). Validation of a phase-mass characterization concept and interface for acoustic biosensors. Sensors.

[42] Chen D., Sun X., Zhang K., Fan G., Wang Y., Li G., Hu R. (2017). A Noncontact dibutyl phthalate sensor based on a wireless-electrodeless QCM-D modified with nano-structured nickel hydroxide. Sensors.

[43] Ogi H. (2013). Wireless-electrodeless quartz-crystal-microbalance biosensors for studying interactions among biomolecules: A review. Proc. Jpn. Acad. Ser. B Phys. Biol. Sci..

[44] Ogi H., Fukunishi Y., Nagai H., Okamoto K., Hirao M., Nishiyama M. (2009). Nonspecific-adsorption behavior of polyethylenglycol and bovine serum albumin studied by 55-MHz wireless-electrodeless quartz crystal microbalance. Biosens. Bioelectron..

[45] Ogi H., Motohisa K., Hatanaka K., Ohmori T., Hirao M., Nishiyama M. (2007). Concentration dependence of IgG-protein A affinity studied by wireless-electrodeless QCM. Biosens. Bioelectron..

[46] Ogi H., Yanagida T., Hirao M., Nishiyama M. (2011). Replacement-free mass-amplified sandwich assay with 180-MHz electrodeless quartz-crystal microbalance biosensor. Biosens. Bioelectron..

[47] Huang X., Bai Q., Hu J., Hou D. (2017). A practical model of quartz crystal microbalance in actual applications. Sensors.

[48] Edvardsson M., Rodahl M., Kasemo B., Höök F. (2005). A dual-frequency QCM-D setup operating at elevated oscillation amplitudes. Anal. Chem..

[49] Tembhare P.C., Rangaree P.H. (2017). A review on: Design of 2.4GHz FBAR filter using MEMS technology for RF applications. Imp. J. Interdiscip. Res..

[50] Fu Y.Q., Luo J.K., Nguyen N.T., Walton A.J., Flewitt A.J., Zu X.T., Li Y., McHale G., Matthews A., Iborra E. (2017). Advances in piezoelectric thin films for acoustic biosensors, acoustofluidics and lab-on-chip applications. Prog. Mater. Sci..

[51] Colangeli L., Lopez-Moreno J.J., Nørnberg P., Della Corte V., Esposito F., Mazzotta Epifani E., Merrison J., Molfese C., Palumbo P., Rodriguez-Gomez J.F. (2009). MEDUSA: The ExoMars experiment for in-situ monitoring of dust and water vapour. Planet. Space Sci..

[52] Battaglia R., Palomba E., Palumbo P., Colangeli L., Della Corte V. (2004). Development of a micro-balance system for dust and water vapour detection in the Mars atmosphere. Adv. Space Res..

[53] Elam J.W., Pellin M.J. (2005). GaPO_4_ sensors for gravimetric monitoring during atomic layer deposition at high temperatures. Anal. Chem..

[54] Thanner H., Krempl P.W., Wallnöfer W., Worsch P.M. (2002). GaPO_4_ high temperature crystal microbalance with zero temperature coefficient. Vacuum.

[55] Devine T.M., Chakravarti R., Patrick B.N., Giheny K.P.E. (2016). Method and Application of GaPO_4_ Crystal Microbalance to High Acid Crude Corrosion Testing. U.S. Patent.

[56] Jakab S., Picart S., Tribollet B., Rousseau P., Perrot H., Gabrielli C. (2009). Study of the dissolution of thin films of cerium oxide by using a GaPO_4_ crystal microbalance. Anal. Chem..

[57] Millichamp J., Ali E., Brandon N.P., Brown R.J.C., Hodgson D., Kalyvas C., Manos G., Brett D.J.L. (2011). Application of a GaPO_4_ crystal microbalance for the detection of coke formation in high-temperature reactors and solid oxide fuel cells. Ind. Eng. Chem. Res..

[58] Thanner H., Krempl P.W., Selic R., Wallnöfer W., Worsch P.M. (2003). GaPO_4_ high temperature crystal microbalance demonstration up to 720 °C. J. Therm. Anal. Calorim..

[59] Zu H., Wu H., Wang Q.M. (2016). High-temperature piezoelectric crystals for acoustic wave sensor applications. IEEE Trans. Ultrason. Ferroelectr. Freq. Control.

[60] Cernosek R.W., Martin S.J., Hillman A.R., Bandey H.L. Comparison of lumped-element and transmission-line models for thickness-shear-mode quartz resonator sensors. Proceedings of the IEEE International Frequency Control Symposium.

[61] Arbuckle-Keil G.A. (2002). The quartz crystal microbalance in electrochemistry. Characterization of Materials.

[62] Martin S.J., Granstaff V.E., Frye G.C. (1991). Characterization of a quartz crystal microbalance with simultaneous mass and liquid loading. Anal. Chem..

[63] Arnau A. (2008). A review of interface electronic systems for AT-cut quartz crystal microbalance applications in liquids. Sensors.

[64] Rodahl M., Höök F., Kasemo B. (1996). QCM operation in liquids:  An explanation of measured variations in frequency and Q factor with liquid conductivity. Anal. Chem..

[65] Auge J., Hauptmann P., Eichelbaum F., Rösler S. (1994). Quartz crystal microbalance sensor in liquids. Sens. Actuators B Chem..

[66] Chen Q., Xu S., Liu Q., Masliyah J., Xu Z. (2016). QCM-D study of nanoparticle interactions. Adv. Colloid Interface Sci..

[67] Huang X., Bai Q., Zhou Q., Hu J. (2017). The resistance-amplitude-frequency effect of in—Liquid quartz crystal microbalance. Sensors.

[68] Johannsmann D. (2015). The Quartz Crystal Microbalance in Soft Matter Research.

[69] Saha T., Guo N.Q., Ramakrishnan N. (2016). Zinc oxide nanostructure-based langasite crystal microbalance ultraviolet sensor. IEEE Sens. J..

[70] Astrid P., Arne L., Diethelm J. (2015). Coupled resonances allow studying the aging of adhesive contacts between a QCM surface and single, micrometer-sized particles. Nanotechnology.

[71] Furusawa H., Sekine T., Ozeki T. (2016). Hydration and viscoelastic properties of high- and low-density polymer brushes using a quartz-crystal microbalance based on admittance analysis (QCM-A). Macromolecules.

[72] Wudy F., Multerer M., Stock C., Schmeer G., Gores H.J. (2008). Rapid impedance scanning QCM for electrochemical applications based on miniaturized hardware and high-performance curve fitting. Electrochim. Acta..

[73] Mills C.A., Chai K.T.C., Milgrew M.J., Glidle A., Cooper J.M., Cumming D.R.S. (2006). A multiplexed impedance analyzer for characterizing polymer-coated QCM sensor arrays. IEEE Sens. J..

[74] Kang Q., Qi Y., Zhang P., Shen D. (2007). Improve the signal-to-noise ratio of a quartz crystal microbalance in an impedance analysis method. Talanta.

[75] Torres R., García J.V., Arnau A., Perrot H., Kim L.T.T., Gabrielli C. (2008). Improved frequency/voltage converters for fast quartz crystal microbalance applications. Rev. Sci. Instrum..

[76] Marco F., Vittorio F. (2009). An oscillator circuit for dual-harmonic tracking of frequency and resistance in quartz resonator sensors. Meas. Sci. Technol..

[77] Cassiède M., Paillol J.H., Pauly J., Daridon J.L. (2010). Electrical behaviour of AT-cut quartz crystal resonators as a function of overtone number. Sens. Actuators A Phys..

[78] Kasper M., Traxler L., Salopek J., Grabmayr H., Ebner A., Kienberger F. (2016). Broadband 120 MHz impedance quartz crystal microbalance (QCM) with calibrated resistance and quantitative dissipation for biosensing measurements at higher harmonic frequencies. Biosensors.

[79] Zhou J.-P., Bao Y., Lin Q., Pang R.-S., Wang L.-M., Niu L. (2014). A new quartz crystal microbalance measuring method with expansive frequency range and broadband adaptive response capacity. Chin. J. Anal. Chem..

[80] Kankare J., Loikas K., Salomäki M. (2006). Method for measuring the losses and loading of a quartz crystal microbalance. Anal. Chem..

[81] Yuan Y.J., Liang T., Han K. (2016). Development of a real-time QCM bond-rupture system for POCT applications. IEEE Sens. J..

[82] Antonio A., Yeison M., José V.G., Yolanda J. (2009). A different point of view on the sensitivity of quartz crystal microbalance sensors. Meas. Sci. Technol..

[83] Chagnard C., Gilbert P., Watkins A.N., Beeler T., Paul D.W. (1996). An electronic oscillator with automatic gain control: EQCM applications. Sens. Actuators B Chem..

[84] Chao Z., Guanping F. (1996). Contributions of amplitude measurement in QCM sensors. IEEE Trans. Ultrason. Ferroelectr. Freq. Control.

[85] Matthys R.J. (1992). Crystal Oscillator Circuits.

[86] Barnes C. (1991). Development of quartz crystal oscillators for under-liquid sensing. Sens. Actuators A Phys..

[87] Barnes C. (1992). Some new concepts on factors influencing the operational frequency of liquid-immersed quartz microbalances. Sens. Actuators A Phys..

[88] Arnau A., García J.V., Jimenez Y., Ferrari V., Ferrari M. (2008). Improved electronic interfaces for AT-cut quartz crystal microbalance sensors under variable damping and parallel capacitance conditions. Rev. Sci. Instrum..

[89] Arnau A., Sogorb T., Jimenez Y. (2001). A new method for continuous monitoring of series resonance frequency and simple determination of motional impedance parameters for loaded quartz-crystal resonators. IEEE Trans. Ultrason. Ferroelectr. Freq. Control.

[90] Arnau A., Jimenez Y., Sogorb T. (2002). Circuit for continuous monitoring of quartz-crystal resonators in sensor applications. Electron. Lett..

[91] Sell J.K., Niedermayer A.O., Jakoby B. (2011). A digital PLL circuit for resonator sensors. Sens. Actuators A Phys..

[92] Koçum C., Erdamar A., Ayhan H. (2009). Design of temperature controlled quartz crystal microbalance system. Instrum. Sci. Technol..

[93] OpenQCM. OpenQCM Electronics Circuit—Pierce Oscillator.

[94] Satoh T., Ruslan R.I., Gotoh S., Akitsu T. (2011). Double-resonance quartz crystal oscillator and excitation of a resonator immersed in liquid media. IEEE Trans. Ultrason. Ferroelectr. Freq. Control.

[95] Ruslan R.I.B., Satoh T., Akitsu T. (2012). Voltage-controlled double-resonance quartz oscillator using variable-capacitance diode. IEEE Trans. Ultrason. Ferroelectr. Freq. Control.

[96] Rodriguez-Pardo L., Farina J., Gabrielli C., Perrot H., Brendel R. (2007). Design considerations of miller oscillators for high-sensitivity QCM sensors in damping media. IEEE Trans. Ultrason. Ferroelectr. Freq. Control.

[97] Rodriguez-Pardo L., Rodriguez J.F., Gabrielli C., Perrot H., Brendel R. (2008). TSM-AW sensors based on miller XCOs for microgravimetric measurements in liquid media. IEEE Trans. Instrum. Meas..

[98] García-Martinez G., Bustabad E.A., Perrot H., Gabrielli C., Bucur B., Lazerges M., Rose D., Rodriguez-Pardo L., Fariña J., Compère C. (2011). Development of a mass sensitive quartz crystal microbalance (QCM)-based DNA biosensor using a 50 MHz electronic oscillator circuit. Sensors.

[99] Rodriguez-Pardo L., Farina J., Gabrielli C., Perrot H., Brendel R. Methodology of design of electronic circuit oscillators for QCM sensors in liquid media. Proceedings of the IEEE International Frequency Control Symposium and Exposition.

[100] Rodriguez-Pardo L., Cao-Paz A., Fariña J. (2011). QCM oscillator sensors: Comparison between miller and ABO topologies. Procedia Eng..

[101] Pinheiro A.C., Bourbon A.I., Cerqueira M.A., Maricato É., Nunes C., Coimbra M.A., Vicente A.A. (2015). Chitosan/fucoidan multilayer nanocapsules as a vehicle for controlled release of bioactive compounds. Carbohydr. Polym..

[102] Muckley E.S., Lynch J., Kumar R., Sumpter B., Ivanov I.N. (2016). PEDOT: PSS/QCM-based multimodal humidity and pressure sensor. Sens. Actuators B Chem..

[103] Liu N., Han J., Liu Z., Qu L., Gao Z. (2013). Rapid detection of endosulfan by a molecularly imprinted polymer microsphere modified quartz crystal microbalance. Anal. Methods.

[104] Trotochaud L., Young S.L., Ranney J.K., Boettcher S.W. (2014). Nickel—Iron oxyhydroxide oxygen-evolution electrocatalysts: The role of intentional and incidental iron incorporation. J. Am. Chem. Soc..

[105] Beißner S., Thies J.W., Bechthold C., Kuhn P., Thürmann B., Dübel S., Dietzel A. (2017). Low-cost, in-liquid measuring system using a novel compact oscillation circuit and quartz-crystal microbalances (QCMs) as a versatile biosensor platform. J. Sens. Sens. Syst..

[106] Jakoby B., Art G., Bastemeijer J. (2005). Novel analog readout electronics for microacoustic thickness shear-mode sensors. IEEE Sens. J..

[107] Hu Z., Hedley J., Keegan N., Spoors J., Gallacher B., McNeil C. (2016). One-port electronic detection strategies for improving sensitivity in piezoelectric resonant sensor measurements. Sensors.

[108] Arnau A., Garcia J.V., Jimenez Y., Ferrari V., Ferrari M. Improved electronic interfaces for heavy loaded AT cut quartz crystal microbalance sensors. Proceedings of the IEEE International Frequency Control Symposium Joint with the 21st European Frequency and Time Forum.

[109] Narbón J.V.G. (2016). Improved Characterization Systems for Quartz Crystal Microbalance Sensors: Parallel Capacitance Compensation for Variable Damping Conditions and Integrated Platform for High Frequency Sensors in High Resolution Applications. Ph.D. Thesis.

[110] Rodahl M., Höök F., Krozer A., Brzezinski P., Kasemo B. (1995). Quartz crystal microbalance setup for frequency and Q-factor measurements in gaseous and liquid environments. Rev. Sci. Instrum..

[111] Rodahl M., Kasemo B. (1996). A simple setup to simultaneously measure the resonant frequency and the absolute dissipation factor of a quartz crystal microbalance. Rev. Sci. Instrum..

[112] Dixon M.C. (2008). Quartz crystal microbalance with dissipation monitoring: Enabling real-time characterization of biological materials and their interactions. J. Biomol. Tech..

[113] Rodahl M., Höök F., Krozer A., Kasemo B. (1996). A Piezoelectric Crystal Microbalance Device. W.O. Patent.

[114] Baù M., Ferrari M., Ferrari V. (2017). Analysis and validation of contactless time-gated interrogation technique for quartz resonator sensors. Sensors.

[115] Ferrari M., Baù M., Pagnoni M., Ferrari V. Compact DDS-based system for contactless interrogation of resonant sensors based on time-gated technique. Proceedings of the IEEE Sensors.

[116] Ferrari M., Baù M., Masud M., Ferrari V. (2016). A Time-gated contactless interrogation system for frequency and quality factor tracking in QCR to investigate on liquid solution microdroplets. Procedia Eng..

[117] Drees D.M., Shanks H.R., Van Deusen R.A., Landin A.R. (1999). Method and System for Detecting Material Using Piezoelectric Resonators. U.S. Patent.

[118] Montagut Y.J., García J.V., Jiménez Y., March C., Montoya A., Arnau A. (2011). Frequency-shift vs. phase-shift characterization of in-liquid quartz crystal microbalance applications. Rev. Sci. Instrum..

[119] March C., García J.V., Sánchez Á., Arnau A., Jiménez Y., García P., Manclús J.J., Montoya Á. (2015). High-frequency phase shift measurement greatly enhances the sensitivity of QCM immunosensors. Biosens. Bioelectron..

[120] Antonio A., Mollá P.G., Narbon J.V.G., Jiménez Y.J., Ferizzola Y.M., Fabado A.R. (2014). Method and Device for Nanogravimetry in Fluid Media Using Piezoelectric Resonators. U.S. Patent.

[121] Ferrari V., Marioli D., Taroni A. (2001). Improving the accuracy and operating range of quartz microbalance sensors by a purposely designed oscillator circuit. IEEE Trans. Instrum. Meas..

[122] Rocha-Gaso M.-I., García J.-V., García P., March-Iborra C., Jiménez Y., Francis L.-A., Montoya Á., Arnau A. (2014). Love wave immunosensor for the detection of carbaryl pesticide. Sensors.

[123] Cao-Paz A., Rodriguez-Pardo L., Fariña J. (2014). Temperature compensation of QCM sensors in liquid media. Sens. Actuators B Chem..

[124] Kakalis A., Panayiotou C. (2017). The temperature effect of AT-cut input quartz parameters on QCM effective properties calculated with equivalent circuit models. J. Electroceram..

[125] Saha T., Guo N., Ramakrishnan N. (2016). A novel langasite crystal microbalance instrumentation for UV sensing application. Sens. Actuators A Phys..

[126] Habuka H., Matsui M. (2013). Langasite crystal microbalance frequency behavior over wide gas phase conditions for chemical vapor deposition. Surf. Coat. Technol..

[127] Nosek J., Pustka M. (2006). Determination of the electromechanical coupling factor of gallium orthophosphate (GaPO_4_) and its influence on resonance-frequency temperature dependencies. IEEE Trans. Ultrason. Ferroelectr. Freq. Control.

[128] Aubert T., Elmazria O., Assouar M.B. Wireless and batteryless surface acoustic wave sensors for high temperature environments. Proceedings of the 9th International Conference on Electronic Measurement & Instruments.

[129] PiezoCryst. High Temperature Microbalance Sensor Crystals: Type R-30.

[130] Millichamp J., Mason T.J., Brandon N.P., Brown R.J.C., Maher R.C., Manos G., Neville T.P., Brett D.J.L. (2013). A study of carbon deposition on solid oxide fuel cell anodes using electrochemical impedance spectroscopy in combination with a high temperature crystal microbalance. J. Power Sources.

